# Identifying significant genetic regulatory networks in the prostate cancer from microarray data based on transcription factor analysis and conditional independency

**DOI:** 10.1186/1755-8794-2-70

**Published:** 2009-12-21

**Authors:** Hsiang-Yuan Yeh, Shih-Wu Cheng, Yu-Chun Lin, Cheng-Yu Yeh, Shih-Fang Lin, Von-Wun Soo

**Affiliations:** 1 Department of Computer Science, National Tsing Hua University, HsinChu 300, Taiwan; 2 Institute of Information Systems and Applications, National Tsing Hua University, HsinChu 300, Taiwan; 3 Department of Computer Science and Information Engineering, National University of Kaohsiung, Kaohsiung 811, Taiwan

## Abstract

**Background:**

Prostate cancer is a world wide leading cancer and it is characterized by its aggressive metastasis. According to the clinical heterogeneity, prostate cancer displays different stages and grades related to the aggressive metastasis disease. Although numerous studies used microarray analysis and traditional clustering method to identify the individual genes during the disease processes, the important gene regulations remain unclear. We present a computational method for inferring genetic regulatory networks from micorarray data automatically with transcription factor analysis and conditional independence testing to explore the potential significant gene regulatory networks that are correlated with cancer, tumor grade and stage in the prostate cancer.

**Results:**

To deal with missing values in microarray data, we used a K-nearest-neighbors (KNN) algorithm to determine the precise expression values. We applied web services technology to wrap the bioinformatics toolkits and databases to automatically extract the promoter regions of DNA sequences and predicted the transcription factors that regulate the gene expressions. We adopt the microarray datasets consists of 62 primary tumors, 41 normal prostate tissues from Stanford Microarray Database (SMD) as a target dataset to evaluate our method. The predicted results showed that the possible biomarker genes related to cancer and denoted the androgen functions and processes may be in the development of the prostate cancer and promote the cell death in cell cycle. Our predicted results showed that sub-networks of genes SREBF1, STAT6 and PBX1 are strongly related to a high extent while ETS transcription factors ELK1, JUN and EGR2 are related to a low extent. Gene SLC22A3 may explain clinically the differentiation associated with the high grade cancer compared with low grade cancer. Enhancer of Zeste Homolg 2 (EZH2) regulated by RUNX1 and STAT3 is correlated to the pathological stage.

**Conclusions:**

We provide a computational framework to reconstruct the genetic regulatory network from the microarray data using biological knowledge and constraint-based inferences. Our method is helpful in verifying possible interaction relations in gene regulatory networks and filtering out incorrect relations inferred by imperfect methods. We predicted not only individual gene related to cancer but also discovered significant gene regulation networks. Our method is also validated in several enriched published papers and databases and the significant gene regulatory networks perform critical biological functions and processes including cell adhesion molecules, androgen and estrogen metabolism, smooth muscle contraction, and GO-annotated processes. Those significant gene regulations and the critical concept of tumor progression are useful to understand cancer biology and disease treatment.

## Background

Prostate cancer is a leading cancer and aggressive metastasis disease worldwide and it is the second common cancer-death among men [1]. According to the clinical heterogeneity, prostate cancer displays different behaviors related to aggressive metastasis disease. Some experiments discovered that high Gleason grade and advanced pathological stage tumours associated with cancer recurrence tend to have higher aggressive cancer [2]. Currently, prognostication and treatment are based on the clinical stage and Gleason stage but the gene regulation and biological processes correlated to the progression of the prostate cancer are still unclear.

The recent microarray technology provides a large-scale measurement of expressions of thousands of genes and uses to manifest the expressions of genes in a particular cell type of an organism at a particular time under particular conditions. This high-throughput experimental technology is a powerful tool for comparing mutant or diseased cells with normal cells and searching for differences in gene expressions that can be the potential key factors leading to diseases. Several studies use the wet-lab experiments and microarray data analysis to detect strong significant genes as markers from gene expression level. Although microarray studies of prostate cancer have already identified the different gene expressions between normal and cancer, they still use the traditional unsupervised clustering methods to realize the potential molecular variation with individual genes. However, microarray data reveals information related to not only gene expressions but also to genetic networks of biological experiments or in vivo screen examinations. The general purpose of inferring genetic regulatory network is to extract the expression features, activations and inhibitions from the changes of gene expressions among those genes in microarray data. Recently, researches study the reverse engineering methods and try to understand the complex interactions that are directly affected by the genetic networks. Several mathematical methods for modelling the genetic networks have been proposed such as Boolean networks [3], differential equations [4], Bayesian networks [5], and Petri Net [6]. Although they could successfully model the networks to some extent for each gene, it is in general difficult to determine the correct interactions among genes without involving the detailed biological knowledge about their DNA sequences and transcription factors. There are two approaches can be used for learning the popular-used Bayesian networks from data [7] and both two approaches have their advantages and disadvantages. The first one is searching and scoring method, which computes the conditional probability of each network given the data, ranks the networks and searches the best network that can fit the data. The advantage of this approach is the result of network graph with fine-grained probabilistic information but the drawback of this approach is the number of possible networks becomes super-exponential when the number of nodes is very large. Because this approach is NP-hard, the search heuristics method must be adopted. The second approach is constraint-based learning method which uses a different viewpoint to learn the network from data. The basic idea to construct a network is based on the conditional dependencies among nodes given the data. The approach tries to discover all the conditional independencies from data and uses these conditional independencies (CI) to infer the networks. Since the constraint-based learning method needs to get all the conditional independencies which are developed to measure the relationship of dependencies, it is also a hard work to generate the while possible assembling patterns among genes in the microarray data.

However, gene networks inferred solely based on the microarray data are often not sufficient for rigorous analysis. A common problem in such kind of data-driven learning approaches is that only a small number of genes can be modelled. Without sufficient background knowledge supported, it is hard to reconstruct gene regulatory networks merely based on Bayesian learning from scarce data. To overcome the problem, integrate the biological knowledge into the modelling process becomes necessary [8-11]. In molecular biology, biologists believe the expressions of the genes are always controlled by the transcription factors that leads to gene expression change observed in microarray data. Therefore networks between the transcription factors and their target genes are important in understanding the complex regulatory mechanisms in a cell.

Our original idea is to develop an initial gene network combining independency test and transcription factor analysis from the microarray data. We revise and infer the gene networks using d-separation criteria and conditional independency for the direct or indirect interactions in the network. Many biological databases and information services are also available on web browsers via internet and they allow us to gather information about the biological sequences and predict their functionalities and promoter regions to some extent. We apply web services technology to integrate all tools and databases developed by ourselves and others to automatically carry out the workflow of all tasks needed in the computational analysis.

## Methods

**Figure 1:**
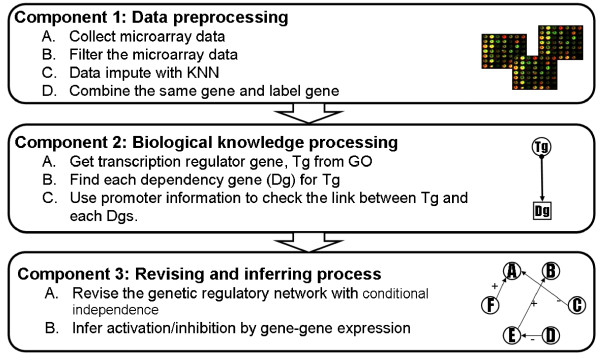
**Overview of the system architecture**.

Our system consists of three main modules: (i) Microarray data pre-processing, (ii) Transcription factor analysis, and (iii) Revising gene network based on conditional independency. Figure 1 shows a workflow of the three major modules in our systems and the steps in each module. Module I deals with the problem of missing values in the microarray using the K-nearest-neighbour (KNN) approach. Module II uses transcription factor analysis to construct an initial regulatory genetic network. And module III revises the genetic regulatory network constructed from component II using d-separation criteria to test conditional independency among genes.

### Cope with missing values in Microarray data

The microarray dataset consists of N genes and M experiments can be represented as an M*N matrix. It presents different gene expression levels X_ij _(i ∈ M, j ∈ N) in this matrix. Gene expressions (either over-expressed or under-expressed) can be revealed in terms of two colors in the microarray data with the symbol "R" representing the red dye; whereas the symbol "G" representing the green dye. The ratios between the two colors reflect the relative degrees of expressions of genes. We extract the data Log_2 _[R/G Normalized Ratio (Medium)] of each gene because the mean value of the normalized ratio is much easier to be affected by noise than the medium value.

Although microarray can be used to detect thousands of genes under a variety of conditions, there are still many missing values in microarray [12]. The reasons for missing values include insufficient resolution, image corruption, and dust or scratches on the slide. If a gene contains many missing values in experiments, it is not easy to determine a precise expression value for each gene that causes a difficulty in the subsequent analysis of the regulation networks. However, we can not simply remove all gene data that contains missing values because the number of remaining genes will become too small to predict the network correctly. In order to get a better result, the genes that contain less than 20% entries missing in all experiment are picked. In order to get as complete data as possible, we use the K-Nearest-Neighbors (KNN) algorithm [12] to estimate the missing values. Suppose there is one missing value of gene A in N samples. The steps of KNN algorithm are listed as follows:

1) We consider gene A with the missing value in experiment *t *and calculate the Euclidean distance between Gene A and other genes without missing values in other *t-1 *experiment. Suppose (p_1_, p_2_, p_t-1_, p_t+1_, ..., p_N_) and (q_1_, q_2_, q_t-1_, q_t+1_,..., q_N_) are the expression values of the gene A and other genes in other N-1 experiments. The Euclidean distance between the two gene expressions is as follows:

If genes with missing values in the experiment, we ignore this experiment for calculating the Euclidean distance formula. Because we keep the genes with less than 20% of missing values, there are not many missing data in the microarray data.

2) Select k most similar genes with Euclidean distance to impute missing expression values.

3) Consider the Euclidean distance as weights to average the expression values of k genes.

Supposed D_AB _means the Euclidean distance between Gene A and Gene B and k genes are selected to estimate the missing expression value for Gene A. The weight of gene is:

Given an example in Table 1, Gene_C _has a missing value in sample 1 and we compute D_AC _= 4.06, D_BC _= 3.24, and D_DC _= 6.94. Suppose k = 2, we will select 2 genes that are similar to Gene_C_. Since both D_AC _and D_BC _are smaller than D_DC_, we selected Gene_A _and Gene_B _to impute the missing value of Gene_C _in Table 1. The missing value for Gene_C _s is calculated as follow:

**Table 1:** Gene expressions of the microarray experiment

Gene	E1	E2	E3	E4	E5
Gene_A_	-1.0	-2.0	1.0	-2.0	1.5

Gene_B_	-1.0	-1.0	2.0	1.5	-1.0

Gene_C_	?	-2.0	1.5	2.0	2.0

Gene_D_	-2.0	3.0	4.0	-2.0	3.0

E_i _= the number i of sample in the experiments

In particular, we transformed the continuous expression levels into discrete expression to determine the under-expression and over-expression of genes. The expression values of genes can be separated into two binary values: positive (+) and negative (-). We set reference expression value as the average expression value from all expressions of genes in cancer and normal microarray data [5]. If the gene expression value X_ij _greater than the reference expression value, we regarded as positive (+); else, we regarded as negative (-), respectively. In our experiment, we set -0.06 as reference value.

### Constructing initial gene networks by transcription factor analysis

**Figure 2:**
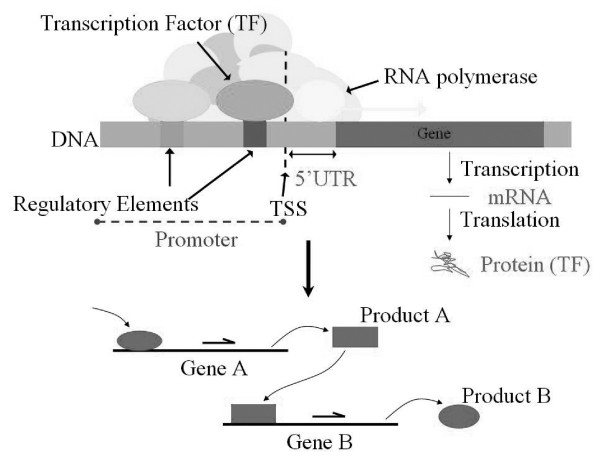
**Transcription regulatory gene and its dependent gene**. Gene A affects the expression of the target gene, Gene B.

Every cell in an organism contains the entire genome which is subdivided into a set of chromosomes and the chromosome is a linear molecule called DNA that is functionally divided into information units called genes. Each gene carries information for the production of a set of proteins which perform a specialized function in the cell. The gene expression is a biological process which converts gene's DNA sequence into its corresponding functional proteins in the cell. A gene is said to be expressed in a cell if its corresponding proteins are present and it can be divided into two regions: a coding region and a regulatory region. The coding region of the gene can be translated into a protein and the regulatory region is the binding site also called promoter region on which a transcription factor can bind. A transcription factor is a protein that can bind on the upward stream of transcription start site (TSS) of the gene in the DNA sequence. Different transcription factors bind on the promoter region will trigger the downstream translation processes. Hence the transcription factors can either enhance or repress the gene expression. For example, in Figure 2, the product of gene A is a transcription factor, which can bind on the promoter region of gene B in the DNA sequence and gene A can affect the expression of its target gene, gene B. A gene that can regulate other genes by its corresponding transcription factor is considered as a transcription regulator gene. In order to construct the initial genetic networks from the transcription factor, we take each gene with the term "transcription regulator activity" specified in Gene Ontology (GO) [13] which contains over 19,000 terms applicable to a wide variety of biological organisms. And then, we use the statistical hypothesis testing to check if there is a link dependency between the transcription regulator gene and other genes in terms of the microarray data. If a transcription regulator gene and another gene are dependent, it means there is a relationship between them.

Assume a null hypothesis that one gene and another gene in the microarray data are independent and check if there is enough evidence to prove this hypothesis with statistical p-value testing. P-value is the probability of obtaining a result and shows the truth of the null hypothesis that the result was chance alone. If the significant level is 0.05, the results are only 5% likely to be as extraordinary as the observation, given that the null hypothesis is true. The statistical formula used to test for independency is as follows [14]:

Where

 = the number of times the expression level of gene = a

 = the number of times both the expression levels of gene = a and gene = b respectively.

M = total data.

G^2 ^has the chi-square distribution with appropriate degrees of freedom *f *= (*r*_1_-1)(*r*_2_-1) where r_1_, r_2 _are the number of expression levels of the data spaces.

For example, suppose gene_1 _and gene_2 _each has the space {+,-} in Table 2 and the statistical test formula for independency is calculated as:

**Table 2:** Sample data for independency test

Gene	E1	E2	E3	E4	E5	E6	E7	E8
Gene_A_	+	+	-	-	-	-	+	-

Gene_B_	-	+	+	-	+	+	-	-

Ei = the number i of sample in the experiment

The degrees of freedom = (2-1)(2-1) = 1 and thus the data has a chi-square distribution with 1 degree of freedom. There are just two variables to do one hypothesis independent testing with chi-square method and the significant p-value is still 0.05. The p-value is calculated as *P*(*U *> .54) ≅ .47. Because this p-value is larger than 0.05, we cannot reject the hypothesis that gene_1 _and gene_2 _are independent. If p-value is less than 0.05, there is enough evidence to conclude gene_1 _and gene_2 _are not independent. We use a pair of genes as an individual independent testing and we do not perform Bonferroni correction to reconstruct the networks. Because the large amount of genes and lots of permutations, the appropriate p-value calculated by Bonferroni correction is too small and conservative. Therefore by using statistical hypothesis testing for a transcription regulator gene against all other genes in the microarray, we could obtain a set of candidate dependent genes with the transcription regulator gene.

According to the independent test, we got the statistical relationships between transcription regulator genes and its dependent genes. Based on biological knowledge, we need to check the link between transcription regulator gene and its dependent gene if the transcription regulator gene's product can bind on the promoter of its dependent gene or not. The internet provides several bioinformatics toolkits that can help us to do the interaction checking but those interfaces are designed to be accessed by humans, not by machines and biologists usually have to spend a lot of time to find, understand and execute the desired computational analysis tools. So, we wrapped the necessary bioinformatics tools as web services and compose the web services into our workflow of interaction checking. Table 3 shows the bioinformatics tools and their web sites we have wrapped. The ExPASy [15] (Expert Protein Analysis System) proteomics server of the Swiss Institute of Bioinformatics (SIB) provides the information of genes. Ensembl [16] is a joint project between EMBL-EBI and the Sanger Institute that develops a software system conducting automatic annotation on selected eukaryotic genomes. TFSEARCH [17] is used to search the transcription factor binding sites and it contains factors of eukaryotic cells from yeasts, plants, arthropods and vertebrates, and position-specific score matrices (PSSM) of the factors to their cis-elements generated from in vitro studies or compiled sites of genes. Matrices from TFSEARCH enable computational prediction of the joining of transfactors and cis-elements in the upstream region of selected genes. In order to automatically selecting and executing the bioinformatics toolkits, we annotated web service in OWL-S functional profiles in Table 4 which is a Semantic Web Service to describe inputs, outputs, preconditions and effects of each web services to compose into a workflow. The functional description of service "inputs" and "outputs" specifies the inputs required by a service and the outputs generated. It also provides the "precondition" to describe the external conditions to be satisfied and the "effects" to describe the expected effects that might result from the execution of a service.

**Table 3:** Bioinformatics tools been wrapped

Tool	Website
TFSEARCH	http://www.cbrc.jp/research/db/TFSEARCH.html

Ensembl	http://www.ensembl.org/index.html

ExPASy	http://au.expasy.org

**Table 4:** The functional profiles of each tool

Tool IO	TFSEARCH	Ensembl	ExPASy
Input	promoter DNA sequence	Gene name	Gene name

Output	Transcription factor	promoter DNA sequence	Transcription factor

**Figure 3:**
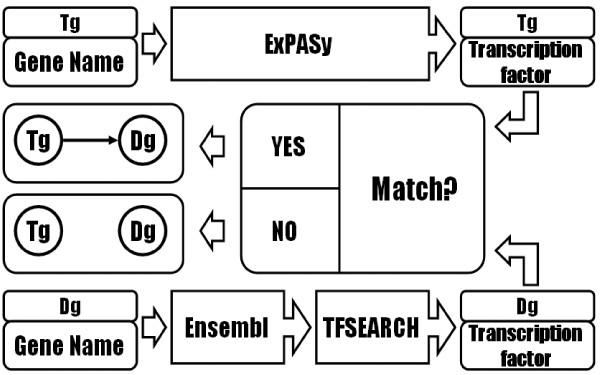
**A workflow of transcription factor analysis**.

Genetic mutation or genomic segments on DNA sequence is one of the reasons in cancer development and the change of genotype in each human may be different, it is hard to extract those sequences to do the analysis. The abnormal relationship (activation or inhibition) or different groups of transcription factors bind on the promoter region also affect the genetic regulation mechanism to present the different phenotype [18]. According to the reason, we use the normal DNA sequence extracted from the public website to predict the possible groups of transcription factors affects dependent gene's transcription from microarray data. First, we pick the 1000 base pair upstream sequence of DNA sequence as promoter region of the dependent gene from Ensembl website and predict all possible transcription factors of the promoter of the gene using TFSEARCH tool. Second, we use ExPASy annotation website to search the transcription factors by the transcription regulator gene. We remove the link between the transcription regulator gene and its dependent gene if there is no transcription factor of the transcription regulator gene can bind on the promoter of its dependent gene. The whole workflow of transcription factor analysis is in Figure 3. Up to this point, we can construct an initial gene regulatory network.

### Revising and inferring the gene networks using conditional independency

The direction of the transcription factor and its dependent gene represents the causal relationship of two nodes in the network structure. We consider the two nodes that are separated by the other nodes and determine whether the relationship between two nodes is direct or indirect. However, if a link connected by a pair of genes not a simple path but also connected by other paths, it is possible that dependency of the pair of genes could not be due to this directed link. Conditional independency test can be used to verify the direct or indirect relationships between the pair of genes when the d-separation set is to be determined [19,20].

**Figure 4:**
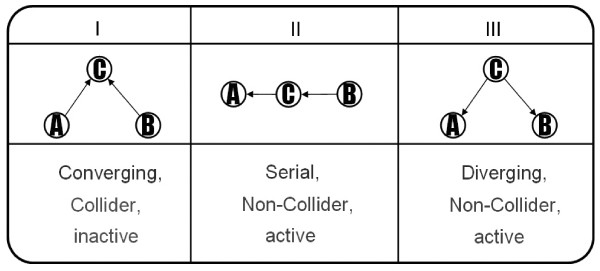
**Example of d-separation concept**.

First, we define three simple types of connections for three nodes on a path as illustrated in Figure 4 to explain the concept of d-separation. In type I, node C serves as a node of converging connection called collider node and nodes A and B have a common effect on node C with no causal connection between them. In the viewpoint of information flow, it shows the information between nodes A and B cannot pass through node C and we also call that the path is inactive as well as a closed path in type I. In type II, a directed path can be found from node B to node A through node C and node B is an indirect cause of node A. In type III, two direct paths are from node C to nodes A and B and node C is a common cause of both two nodes. In the last two types II and III, node C serves as a node of serial and diverging connection called non-collider. If we know the information of node C, the information of node A can be known without node B as well as the information flow between nodes A and B can pass through C and we call those two paths are active. From a causal point of view, nodes A and B are marginally independent if node C is not conditioned but if it is conditioned on C, node A and B are conditionally dependent. Take an example, if it is conditioned on C, the paths in type II and III become blocked and nodes A and B are conditionally independent in which we call those two nodes are d-separated by node C. In addition, the type I path is opened given condition on C.

Since d-separation entails conditional independency, an efficient algorithm for determining whether two nodes are d-separated by a set of nodes is needed. Cheng J. [19] proposed a procedure to solve this problem and found the minimum d-separation set but the execution time of the algorithm grows exponentially with large number of nodes in the network. We use the biological constraints to modify the algorithm and decrease the search space. Given two nodes in the networks as start node and end node, we used depth-first search (DFS) method to traverse all the paths between start and end nodes. While searching the possible paths, we use the following branch-andbound constraints to prune the searching space:

(1) Only transcription regulator gene can link to its dependent genes and transcription regulator gene can be an active node.

(2) If the dependent gene is a collider node in the sub-networks such like type I in Figure 4, the path must be a close path and will be deleted.

**Figure 5:**
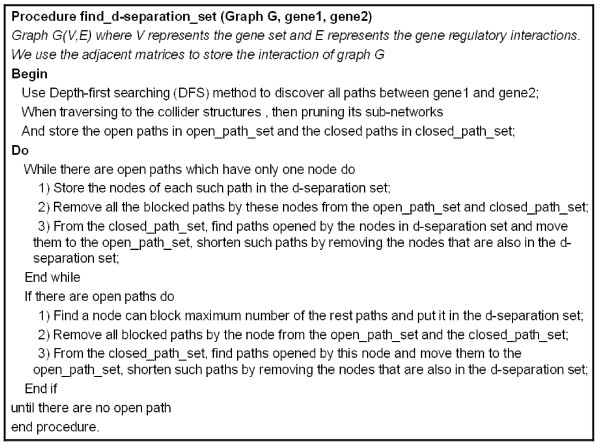
**The algorithm of finding d-separation_set Procedure**.

After finding all non-collider structures between start and end nodes, we rank the candidate d-separation genes by the number of paths they involved in and choose the top one as the d-separate gene that can block a maximum number of paths. Then, we delete all the candidate d-separate genes which are also involved in the same paths with d-separate gene and continue to choose the d-separate gene from the addition candidate d-separation gene sets again until there is no more candidate d-separation genes that can be selected. The detailed procedure is shown in Figure 5.

**Figure 6:**
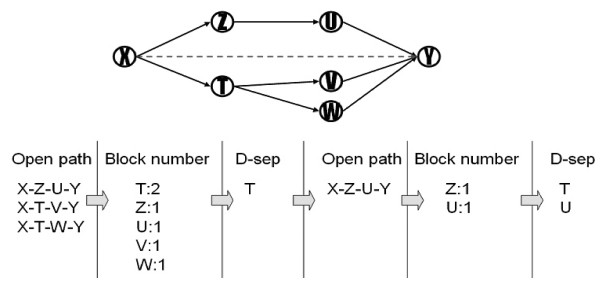
**Example of find_ d-separation_set procedure**. D-sep denotes the d-separate gene.

Take a simple example to explain the d-separation set finding procedure. Assume there are 7 genes in a gene regulatory network that is shown in Figure 6. To check the dependency between genes X and Y, CI test measures the probability of dependency between genes X and Y given by their d-separation set to determine whether there is a directed link between X and Y or not. First, three paths from X and Y are found. They are all open paths and are put into the open-path-set. Then, count the genes in the open-path-set that can block a maximum number of paths. Gene T which blocks two paths (X-T-V-Y and X-T-W-Y) becomes a candidate gene for the d-separation set. After putting T into d-separation set, two paths (X-T-V-Y and X-TW-Y) are removed. There is still one path in the open-path-set, so repeatedly find a gene that can block a maximum number of paths. At this time, genes Z and U both block one path (X-Z-U-Y). Gene U becomes another candidate gene for the dseparation set by a random selection. After path X-Z-U-Y is removed, there are no open paths in the open-path-set and the procedure returns the final d-separation set as {T, U}. Given genes T and U, if X and Y are conditional independent as a result of CI test, the directed links between X and Y are removed.

After finding the minimum d-separate genes between start and end genes, we extend the statistical formula [14] to verify the conditional independency among genes. If we wish to check genes A and B are d-separated by gene C, the following formula is extended to test the conditional independency among genes.

Where

 means the number of times if the expression level of X = a and the expression level of Y = b and the expression level of Z = c

 means the number of times if the expression level of X = a and the expression level of Z = c.

 means the number of times if the expression level of Z = c

The number of degrees of freedom used in the test is 

where r_i _is the number of expression levels of each Xi's space.

For the traditional constraint-based method, it is no way to avoid an exponential number on CI tests for every pair of nodes to make sure that the edges should be kept or removed [14]. After we find the minimum d-separating sets, we determine whether an indirect edge between two nodes should be needed and there are repeated tests of conditional independencies given minimum d-separating sets. With the small size of the minimum d-separating sets, we can do the permutation comparisons by applying Bonferroni correction for multiple testing to renew the significant threshold for each of the n individual tests to maintain an experiment-wise error rate. Comparing with the whole nodes in the network, it is a small set of nodes should be tested in conditional independent testing with Bonferroni correction. Take an example in Figure 6, we want to verify the direct link between node X and Y should be deleted or not in the sub-network. We use the procedure in Figure 5 to extract the minimum dseparate genes, node T and U, to help us determine whether an edge between two nodes should be removed. According to the small size of the d-separating genes, we do the tests in each of the two predicted conditional independence relations as CI(X, Y|T) and CI(X, Y|U) and reject the null hypothesis that both tests are independent with the p-value less than 0.05/2 = 0.025. For different d-separated genes, we can get different significant p-value and so on.

The general purpose of the gene regulatory network analysis is to extract pronounced gene regulatory features (ex. activation and inhibition) by examining gene expression patterns from microarray data. After the network structure has been constructed, the following heuristic rules are used to determine the activation and inhibition relations of the links between two genes, X and Y.

(i) X activates Y: If the expression level of X is over-expressed (+), then Y is also over-expressed; If the expression level of X is under-expressed (-), then Y is also under-expressed.

(ii) X inhibits Y: If the expression level of X is over-expressed (+), then Y is also under-expressed; If the expression level of X is under-expressed (-), then Y is also over-expressed.

However, genes may have inconsistent values across similar samples because the change of environment and some experimental error. The relations of two genes are not always the same in different experiments under the same conditions. In order to determine the relations between two genes with the large number of microarray data supported, we choose the higher number of gene relations in the experiments between pair of genes as the relations based on the heuristic rules. For example, Table 2 shows the binary expression level of gene A and B from 8 microarray experiments. The number of the activation event is 3 and the number of inhibition relation is 5. Because the number of the inhibition relation is higher than the ratio of activation relation, the system identifies the link between gene A and B as inhibition. However, it may hard to determine the relations if the number of action and inhibition is equal. We assume a pair of gene expects to have the same relations under the same condition in microarray data. In our microarray data, there are 66% of the genes with above 80% consistent expression and 99.4% of the genes with above 50% consistent expression across similar samples and more genes with consistent gene expressions will help us to identify the relations between pair of genes correctly.

### Network measure

Some researches [21,22] discover that the gene regulatory networks contain some properties that the links connected with genes non-randomly and we should verify the topology of gene regulatory networks which are reconstructed by our methods. We used the network statistical measure [21] and network motifs [22] to identify the gene network we constructed and extracted the potential genes to compare the sub-networks between the cancer and normal samples. Networks are classified by their degree distributions. The degree of a node is the number of links it connects to other nodes. In the undirected graphs, the average degree <k> is formally defined as following:

*Where l *= the total number of links in the network

N = the total number of vertices in the network

The degree distribution, P(k), is the primary properties of the global architecture of the gene networks. It implies the probability that a selected vertex is connected exactly to k directed neighbours in the network.

Where N(k) = the number of nodes which have k links

N = the total number of vertices in the network

In directed graphs, the in-degree, *k*_*in*_, is the number of incoming edges of the vertex and the out-degree, *k*_*out*_, is the number of outgoing edges of the vertex.

The clustering coefficient measures the tendency of nodes that can form a cluster. C(k) is the average clustering coefficient of all vertices with k links.

Where n = number of triangles that go through the vertex with k links.

K = the number of nearest neighbours of the vertex

The average clustering coefficient measures the overall tendency of nodes that can form clusters. The formula is defined as:

Where the clustering coefficients of all N nodes are averaged over index i.

### Network motifs

**Figure 7:**
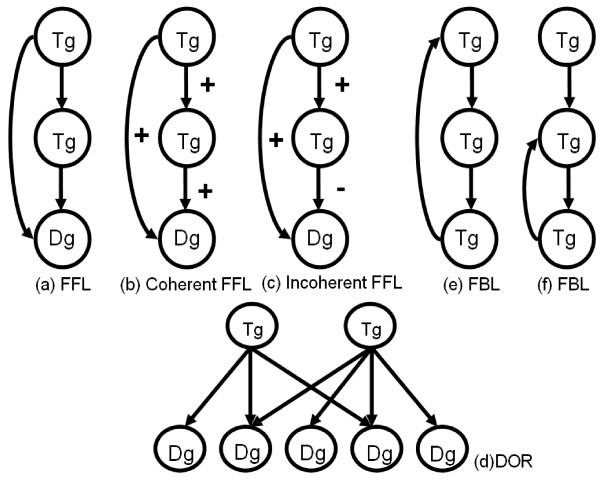
**Network motifs structure**.

Gene regulatory networks may be modelled as all possible interactions among genes. To understand the complex networks, we should look into the networks via simple sub-networks. "Network motifs" describes the frequency patterns of interactions that how genes connect with their neighbours. We discuss three types of network motifs: feed forward loop (FFL), dense overlapping regulons (DOR), and feedback loop (FBL) to compare the cancer and normal gene networks [22]. Feed forward loop (FFL) in Figure 7(a) contains 2 transcription factors and their dependent genes. The first transcription factor regulates the second transcription factor named co-transcription factor, and both transcription factors jointly regulate a dependent gene. The casual relations between each gene can be 'activate' (+) or 'inhibit' (-) in the FFL structure. Therefore, there are eight different structures that can be divided into two types of FFLs: 'coherent FFL' and 'incoherent FFL'. The 'coherent' means the sign of the direct regulation path from the transcription factor to the dependent gene is the same as the overall sign of the indirect regulation path from the transcription factor through the co-transcription factor to the dependent gene in Figure 7(b). For example, the transcription factor and co-transcription factor both activate the dependent genes and the transcription factor also activates co-transcription factor, we call this kind of FFL as coherent. On the other hand, if the transcription factor inhibited the dependent gene, we call it as incoherent FFL as shown in Figure 7(c). The dense overlapping regulons (DOR) represents the overlapping interactions between the groups of transcription factors and their dependent genes in Figure 7(d). We use the clustering method to discover the DOR structure. The dependent genes in the DOR structure which are regulated by a combination of a set of transcription factors that share a common biological function. The Gene Ontology produces thesauri that contain many biological terms organized according to molecular functions, biological processes and cellular components respectively. We use the GO to identify the function of a gene that has a transcription factor regulates in the network. The Feedback loop (FBL) structure contains 3 transcription factors and have loops which are connected the originating and ending point at the same gene. There are two different kinds of FBLs: one is the originating and ending at the same transcription factor as in Figure 7(e) and the other is ending at the co-transcription factor as in Figure 7(f).

## Results

We applied our methods to analyze two microarray datasets: "Gene expression profiling identifies clinically relevant subtypes of prostate cancer" [2]. It consists of 62 primary tumors and 41 normal prostate tissues. The detailed pathological and clinical data are provided in [23]. We extracted the ratio value Log_2 _[R/G Normalized Ratio (Medium)] of each gene by using the normalization function provided by Stanford Microarray Database (SMD).

### Microarray data pre-processing

**Figure 8:**
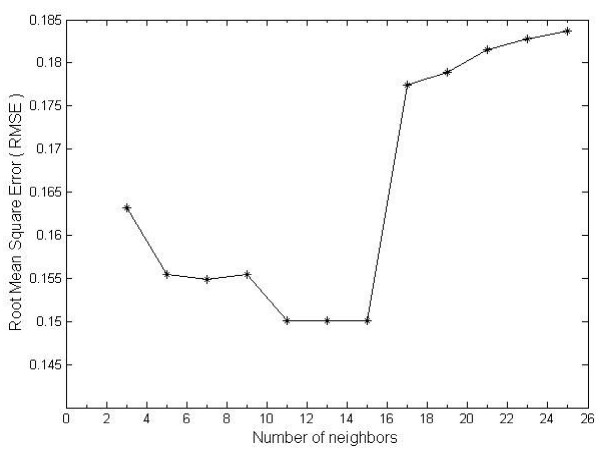
**Effect of number of nearest neighbors used in KNN method for imputing missing values**.

We evaluated the KNN method for imputing missing values in the microarray data. First, we deleted 1,750 original values at random one by one to create test data sets and estimated the missing value to compare with the original value. The accuracy of estimation values are calculated by Root Mean Squared error (RMSE) which sum of the different values between imputed and original values and divided by the number of missing values we computed. The lower RMSE denotes the higher accuracy for estimating the missing values. In Figure 8, it shows that the estimated values with RMSE rates are all under 0.185. While setting the 11-15 nearest neighbors in KNN method, we could get the lower error rate [12]. The number of nearest neighbors in KNN method is higher than 16 or lower than 3 can have greater error rate to estimate the missing values. We extract 15 neighbor genes caused the lower RMSE rate with 20% missing values in the microarray dataset. In microarray data pre-processing, there is detailed statistical information in Table 5. In first column (A), it indicates the number of genes from microarray data and column (B) indicates the number of filter data when we remove the genes that have more than 20% missing values in the microarray dataset. The third column (C) indicates the number of data imputed with KNN (K = 15). From the observation, we filters almost 75% genes from each microarray dataset that means microarray technology can measure thousands of genes simultaneously, but it also contains much noise that causes a lot of missing values. The microarray technology needs to be refined to generate high-quality data so that biologists can identify the gene regulatory relation more precisely. For dealing with missing values in microarry dataset, the overall imputed ratio by KNN algorithmis about 34% see Additional file 1. The imputed ratios of the microarray datasets are all less than 50%, it seems to be reasonable to assume the imputed dataset is good enough to analyze the gene regulatory network. There are 66% of the genes with above 80% consistent expression and 99.4% of the genes with above 50% consistent expression across similar samples and more genes with consistent gene expressions will help us to identify the relations between pair of genes correctly.

**Table 5:** the number of genes in different steps in processing

Step	Cancer	Normal
(A)	44014	44014

(B)	11130	11524

(C)	7588	7673

(A): the number of genes in microarray experimental data

(B): the number of filter data when removing the genes that have more than 20% missing values in the microarray dataset

(C): the number of genes after imputing missing values with KNN (K = 15) method

### Biological knowledge processing

Use the genes after microarray data pre-processing to map 2665 genes that belong to the "transcription regulator activity" category specified in GO. Each gene that can find a match in the category of transcription regulator activity in GO is regarded as a transcription regulator gene. Take an example gene SRF in normal dataset to be treated as a transcription regulator gene and 494 genes are first found to be dependent with SRF using statistical test method. The transcription factor analysis then helps to filter out those links possibly without biological significance and finally resulted in 13 dependency genes that can be considered to "effectively" interact with SRF. Since the biological toolkits and databases are not complete enough, they would tend to miss transcription factors that are not yet found and it may cause the incompleteness of the inferred interaction networks and thus reduces the recall of the inference method that misses some inferred gene relations. But the gene interaction networks found are at least under the sanction of current biological knowledge of transcription factors to the reasonable extent.

### Revising gene regulatory networks based on Bayesian network

We use d-separation concept and conditional independency test after Bonferroni correction to further verify the direct or indirect links between transcription regulator gene and its dependent genes. Table 6 shows the number of links between initial gene regulatory network and the revised gene regulatory network using d-separation concept and conditional independency test. The filter-out ratios of the sub-networks are almost larger than 40% and it means that modelling gene regulatory network only depending on the statistics and biological knowledge between any two genes doesn't fit the real networks inferred from microarray data. It may contain a lot of extra links among genes in the networks. Take gene SRF in normal dataset as a simple example to show the CI test between SRF and its transcription factor dependent genes in Table 7. Row "Dg of SRF" shows 7 transcription factor dependent genes of SRF. Row "d-sep genes" denotes the dseparation set between SRF and each dependent gene by using d-separation set finding procedure. "p-value" shows the significant value computed in the conditional independency between SRF and each dependent gene using CI test. "NF" represents none genes can be found in d-separation set and "NA" represents none available because d-sep genes are empty. The p-values of conditional independency between SRF and Gene C14ORF122, STCH, and MARCKS are larger than 0.05 and the direct connected links between SRF to these genes should be removed. It shows gene SRF does not directly affect these genes and has an indirect relationship through gene HSF2 in the normal microarray dataset.

**Table 6:** Number of links between two networks

Links	Cancer	Normal
Initial gene regulatory network (Before CI test)	15659	3765

Revised gene regulatory network (After CI test without Bonferroni correction)	8393	3497

Revised gene regulatory network (After CI test with Bonferroni correction)	8298	3497

Filter-out ratio	47%	7%

**Table 7:** The p-value and d-separation set of SRF gene and its Transcription factor dependent gene

Dependent gene of SRF	CHST5	C14ORF122	STCH	TMED3	ENPP2	MARCKS	SCD5
d-sep genes	NF	HSF2	HSF2	NF	NF	HSF2	NF

p-value	NA	0.09	0.11	NA	NA	0.09	NA

### Network measure

Complex diseases depend on the altered interactions among multiple genes and different expression change in the critical genes comparing with normal cell. We use two points of view to see the different between normal and cancer network: one is global and the other is detailed. Global point of view provides the network topology approach we mentioned in and overall function and pathway enrichment using DAVID[24] and GSEA[25] toolkits. The detailed can give new and interesting genes involved in the specific network motifs which may relate to the cancer and are often quite subjective.

**Figure 9:**
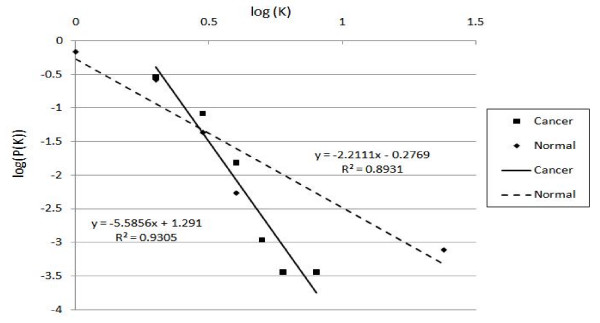
**Degree distribution of cancer and normal on log-log plot e**.

According to the statistical network measure that we mentioned in section 3.4, we use the linear regression to calculate the straight line in a double logarithmic plot that shows the degree distribution against the number of links in Figure 9. While x-axis represents the log of the k links and y-axis represents the log of degree distribution. The linear fitting function of cancer network is y = -5.5856x+1.291 with the correlation R^2 ^= 0.9305, otherwise, the normal network is y = -2.2111x-0.2769 with the correlation R^2 ^= 0.8931. The topology of the degree distribution forms a straight line and it indicates a scale-free connectivity distribution. Scale-free networks have a few nodes with a very large number of links and many nodes with only a few links [21]. Some transcription factors are connected with each other and may play an important role in regulatory events. In our experiment data, R_2 _correlations are all larger than 0.75, it means the relationship fits very well to a linear function. It can be concluded that the topology of gene regulatory networks modeled by our method are actually scale-free networks that are compatible with previous studies regarding the topology property of a gene network We also calculate the probability P(k) of finding a vertex with k links using follows the formula:

where *s *= scaling exponent of the network.

**Figure 10:**
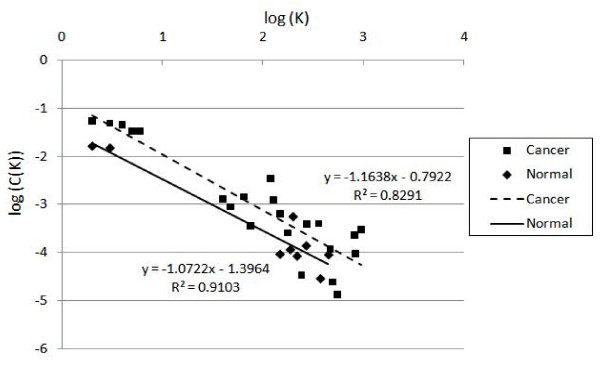
**Clustering coefficients of cancer and normal networks on log-log plot**.

In the normal gene regulatory network, the in-degree of the gene is 1.465216 that means each dependent gene is regulated by less than 2 transcription factors. In the cancer gene regulatory network, the in-degree is 2.84916 means that each gene is regulated by nearly 3 transcription factors. Cancer networks seem to have more complex interactions than normal network. In Figure 10, we use the linear regression to calculate the straight line in a double logarithmic plot for the clustering coefficient against the number of links while x-axis represents the log of the k links and y-axis represents the log of clustering coefficient. The linear fitting function of the regulated network of a normal gene is y = -1.0722x-1.3954 with the correlation R^2 ^= 0.9103. The linear fitting function of the regulated network of a cancer gene is y = -1.1638x-0.7922 with the correlation R^2 ^= 0.8291. The clustering coefficient C(k) depending on the link k can be approximated with a power law formula as follows:

where *w *= scaling exponent of the network

The value of scaling exponent *w *is close to -1 that indicates that the hierarchical modularity and both cancer and normal samples tend to have a hierarchical modular structure. It implies that the sparsely connected nodes in the gene regulatory network are part of highly clustered regions with communication [26]. Table 8 summarizes the comparison on the overall statistical measures of networks for both normal and cancer samples. While the mutation is happened in cancer networks, the overall stability of a network in the biological system should against the changes. The highly connected genes in the network may cause major global effects to their dependent genes and the evolution of biological system should lead to increased stability in order to maintain its robustness [27]. High-degree nodes increase in cancer network may use shorter paths to reach other genes and cancer cell may take different routes to regulate cell growth and cell division toward to the metastasis [28]. We use all pairs shortest path Dijkstra's algorithm[29] to detect the length of any one of gene can link to others see Additional file 2 and it shows the shortest paths are most involved in cancer network and the route in cancer network may be shorter. The cancer has ability to metastasize may inherit the changing of one or groups of transcription regulator genes' gene expressions and trigger different genetically interactions in tumor cell [28,30].

**Table 8:** The parameter of degree distribution and clustering coefficient between normal and cancer

Parameter	Normal	Cancer
Degree distribution		
scaling exponent *s*	1.915916	2.120915
<k>	2.71823	3.026623
*k*_*in*_	1.465216	2.84916
Clustering coefficients		
scaling exponent *w*	-0.93048	-1.396294
<C>	0	0.00576

**Figure 11:**
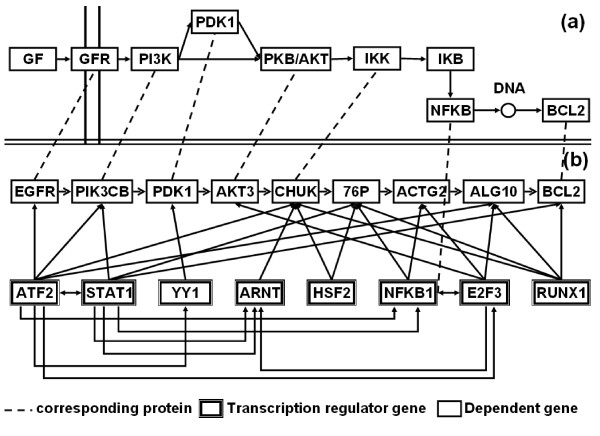
**Reconstruct part of KEGG pathway by our method**.

The mechanism of gene regulations can control the protein interactions of organisms are considered to play fundamental roles in the operation of all processes. We use the synonym names of the dependent gene's product to map the proteins in KEGG pathway[31] and successful reconstruct the paths from EGFR to BCL2 in KEGG pathway database in Figure 11 to validate our results. Figure 11(a) shows the one path in prostate cancer pathways Figure 11(b) shows the paths reconstruct from our method and also provides the upstream of transcription factor genes and their corresponded genes. The dash lines denote the corresponded genes between two paths. We can map the genes in the KEGG path in Figure 11(a) except IKB that did not exist in the microarray data. If pair of the transcription regulator genes has interactions with each other, we can infer their dependent genes also have interactions. For example, transcription regulator gene E2F3 interacts with its dependent gene AKT3 and transcription regulator gene ARNT interacts with its dependent gene CHUK. Because E2F3 interact with ARNT, we can infer the interactions between two dependent genes AKT3 and CHUK and map to their gene products, protein PKB and IKK in prostate cancer pathway.

## Discussion

### Comparison of gene regulatory networks between cancer and normal data

In the cancer and normal network comparison, the transcription regulator genes and their dependent target genes passing through significant p-value using statistical hypothesis testing and promoter analysis. The transcription factors as biomarkers (PBX1, EP300, STAT6, SREBF1, NFKB1, STAT3, EGR1, E2F3, NR2F2) see Additional file 3 are only involved in the cancer networks and those genes are annotated in cancer-related transcription regulatory factors (p-value 1.18E-9). Otherwise, E2F4 only exists in normal network. The regulation of the transcription regulator gene E2F4 plays a key role in the control of normal development and proliferation [32]. 561 extras dependent genes are in normal network; 3495 extras dependent genes are in cancer network and 2,283 genes interact with biomarkers. SREBF1 gene has been shown as up-regulated in the prostate cancer and the early growth response 1 (EGR-1) is a transcription factor regulates the expression of its dependent genes involved in cell growth or survival. We take 2,283 dependent genes affected by biomarkers (PBX1, EP300, STAT6, SREBF1, NFKB1, STAT3, EGR1, E2F3, NR2F2) and not exist in normal network to do the functional annotation using DAVID online toolkit and there are 2,110 genes can be annotated in DAVID toolkit. We filtered the results at least 3 members in each functional category and P-value < 0.05 with Bonferroni correction and FDR<0.25 see Additional file 4. The functional annotation clustering results show that the cancer networks are associated with regulation of progression through cell differentiation, cell death, I-kappaB kinase/NF-kappaB cascade, vesicle-mediated transport, apoptosis biological functions and processes. We also consider performing the pathway enrichment from GSEA online tool which is calculated by hypergeometric distribution method and there are 2,259 genes can be annotated in GSEA online tool. We filtered the indeed functional enrichment canonical pathways from the gene set in our networks with at least 3 members in each functional category and P-value < 0.05. The results denote cell adhesion molecules, androgen and estrogen metabolism, smooth muscle contraction and some GO annotated pathways see Additional file 5. The genes in the cancer network are involved in the significant pathways such as Toll like receptor, PPAR, ERBB, P53 and WNT signaling pathway see Additional file 6. In summary, we have identified androgen related gene TMPRSS2 that is regulated by SREBF1, PBX1 and ETS family members that are associated with the prostate cancer and gene TMPRSS2 has been found in 80% of tumor experiments [33].

**Figure 12:**
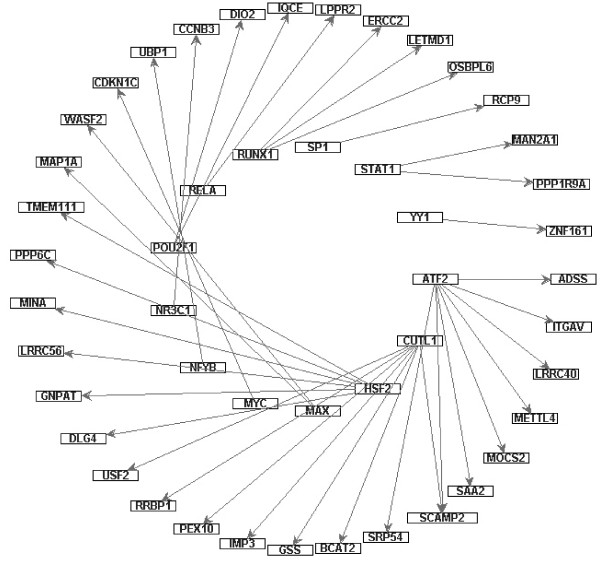
**Transcription regulator genes and their dependent genes which contain the activation interaction in cancer and the inhibition interaction in normal**. Inside circle nodes denote transcription regulator genes and outside circle nodes denote dependent genes.

**Figure 13:**
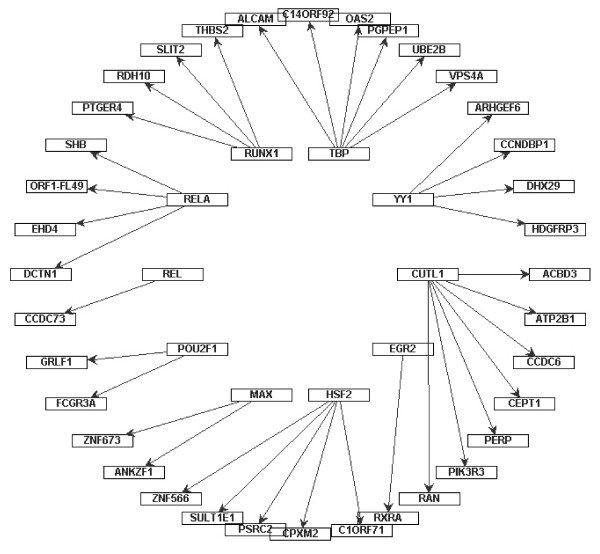
**Transcription regulator genes and their dependent genes which contain the inhibition interaction in cancer and the activation interaction in normal**. Inside circle nodes denote transcription regulator genes and outside circle nodes denote dependent genes.

Besides the difference between the cancer and normal networks, it also consists of interactions of transcription regulator genes and their dependent genes with different expressions between the cancer and normal networks. We take the interactions with the activate expressions in the cancer and the inhibitive expressions in the normal datasets and vice versa. There are 48 directed links that contain 13 transcription regulator genes and each gene in the inside circle is a transcription regulator gene and outside the circle is a transcription factor dependent gene in Figure 12. In addition, there are 36 links that contain 10 transcription regulator genes which contain inhibitive expressions in the cancer and activate expressions in the normal datasets that are shown in Figure 13. See Additional file 7 shows the p-value of the independent test between the transcription regulatory genes and their co-expressed genes with d-separated genes in Figure 12 and 13. Additional file 8 denotes the information of conditional independence testing results between without and with Bonferroni correction in cancer network.

**Figure 14:**
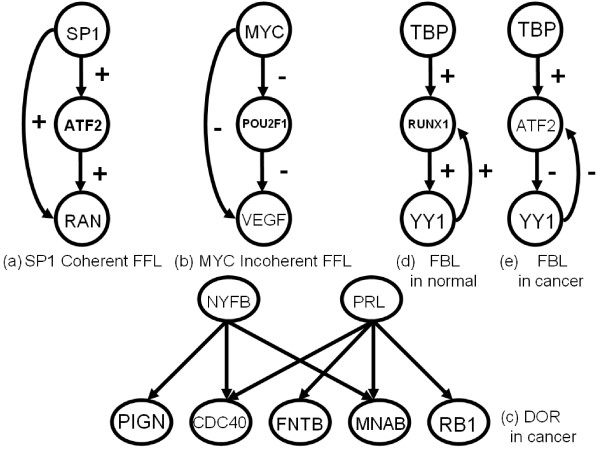
**The example of network motifs in normal and cancer networks**.

If the degree of a vertex is high in the gene networks, it may represent a primary factor in the cancer cell. We combine the degree and different expressions between normal and cancer network in order to identify the potential significant genes. We chose transcription factors YY1, SP1, and MYC in our experiment as an example. Those genes in the cancer network can be mapped to the GO classification that is related to the androgen functions and processes in the development of the prostate cancer and in promoting the cell death in a cell cycle. In the Figure 14(a) shows SP1 has coherent FFLs in cancer gene network. Both SP1 and RAN genes are in the category of positive regulation of transcription and ATF2 gene has a transcription co-activator function in GO respectively. In Figure 14(b) shows that MYC gene in the cancer network decreases the expressions of VEGF and POU2F1 genes and has the negative regulation function. MYC belongs to the cell cycle and apoptosis genes and becomes a key role in developing the prostate cancer. In Figure 14(c), NFYB and PRL genes are identified as having a DOR structure. They both regulate CDC40, FNTB, and MNAB genes. The transcription factor PRL regulates RB1 which has androgen receptor binding function and is involved in the androgen receptor signaling pathway. RB1 gene also has the negative regulation function on cell growth and can promote the cell death. Inhibition of those genes may suggest a possible reason to explain the uncontrolled growth in cancer. In Figure 14(d), TBP is the initial transcription factor and interacts with RUNX1 in the normal gene network. RUNX1. YY1 gene has the feedback loop with RUNX1 gene which belongs to the category of the positive regulation of transcription. We also discover the positive interaction between RUNX1 and YY1 genes. Figure 14(e) shows the feedback loop of YY1 gene in the cancer gene network. Comparing with the normal gene network, YY1 gene shows a different expression level and interacts with ATF2 gene which is mediated by the zinc ion binding of the YY1 molecular function. If zinc function has an early damage, it may lead to a prostate cancer. YY1 gene should play a critical role for the prostate cancer that is reported in [34]. The transcription regulatory genes in FBL, DOR and FFL network motifs in cancer and normal networks see Additional file 9, 10 &11.

For more general evaluation, we used Atlas of Genetics and Cytogenetics in Oncology and Haematology database [35] which collects genes in cancers and divides them into two groups: the annotated match genes and the genes possibly implicated in cancer. There are 37.5% "Match" genes and 62.5% "Possible" genes in our predicted results see Additional file 12 and it also indicate that our method is useful to detect to the possible genes implicated in cancer and the gene regulatory networks constructed by our methods seem to be modelled effectively. Although the verification of the modelling process through literature reports is an indirect way to evaluate gene regulatory networks, it at least shows that the gene regulatory networks modelled by our methods are compatible with the existing literature findings. More detail verifications based on the literature reports see Additional file 13.

### Comparison of gene regulatory networks with different clinical data

Besides comparing the normal and cancer networks, we also identify the significant networks of tumor differentiation at different grades and stages. For the clinical data provided from [2], we divided 62 primary prostate tumor Gleason grade into two classes of low grade and high grade (≦3+4 vs. ≧4+3). It consists of 39 data in low grade class and 23 data in the other class. We detect genes SREBF1, STAT6 and PBX1 that only exist in high grade class and SP1, Elk1, JUN and EGR2 that only exist in low grade class. The expression of ETS transcription factor Elk1 decreases from low grade to high grade samples. We predict the differential expression of several transcription regulator genes (including HSF2, ARNT, MEF2A, ATF2 and YY1) that are strongly related to the cancer grade.

In the other experiments, we divided the pathological stage into two parts, the stage of 28 data belong to early stage (≦T2) and the other 34 data belong to late stage (≧T3). GATA3 regulation happened in the early stage and the outcome of the prostate cancer at the late stage of tumor development that are related to genes MZF1, SREBF1, PRL, and DDIT3. The SP1, MAX, RUNX1 and STAT3 sub-networks are involved in both early and late stages of the tumor but are expressed with different gene expressions. Enhancer of Zeste Homolg 2 (EZH2) expression is regulated by RUNX1, STAT3 and E2F3 and high expression of EZH2 gene is associated with the tumor death and also correlated to the pathological stage [36]. For example, gene SLC22A3 regulated by our predicted significant markers (EP300, STAT6, SRF, PBX1) is strong related in high grade and late stage. The discovery is also reported in most enriched literatures associated with the tumor progression.

## Conclusions

We provide a computational framework to reconstruct the genetic regulatory network from the microarray data using biological knowledge and constraint-based inferences. The method validated in is helpful in verifying possible gene interaction relations in gene networks and filtering out incorrect relations inferred by imperfect methods. We predicted not only individual gene related to cancer but also discovered significant gene regulation networks and the predicted results are also validated in published journals or experiment results. However, to elaborate the work to its best extent, there are still problems to be solved. Since the biological toolkits and databases are not complete enough, they would tend to miss transcription factors that are not yet found. For example, the PSSM from TFSEARCH database is incomplete to detect necessary transcription factors and binding sites. This can reduce the recall of the inference method to miss the inferring genes in the interaction networks. In future work, we could use different microarray data about the cancers to test our methods and integrate further the protein-protein interaction information to construct a more complete gene and protein networks. Then the biologists armed with information of the discover up-stream and down-stream biological interaction mechanisms of genes and proteins could possibly understand more clearly the reaction pathways of biological organisms response to various diseases. We want to explore the network variation underlying different conditions and develop a networkbased method to classify the different clinical heterogeneity.

## Competing interests

The authors declare that they have no competing interests.

## Authors' contributions

HY carried out the design of the workflow, algorithm and molecular studies and drafted the manuscript. SW participated in algorithm design, performed program and statistical analysis. YC focused on design the workflow and algorithm, particular the network analysis and network motif discovery. SF carried out molecular studies and statistical analysis. VW participated in its overall design and coordination of the research and helped to draft the manuscript.

## Pre-publication history

The pre-publication history for this paper can be accessed here:

http://www.biomedcentral.com/1755-8794/2/70/prepub

## Supplementary Material

Additional file 1 **KNN imputed ratio**. Table shows the maximal values can be imputed by KNN algorithm and the number of values is exactly imputed in each microarry data. The imputed ratio is the proportion of the real imputed genes to the maximal genes. The less the imputed ratio is, the more imputed data similar to real experiment data. The result shows that the imputed ratios of the microarray data are all less than 50%, it seems to be reasonable to assume the imputed dataset is good enough to analyze the gene regulatory network. Click here for file

Additional file 2 **All pair shortest path in cancer and normal network**. We use all pairs shortest path Dijkstra's algorithm to detect the length of any one of gene link to other genes in cancer and normal network. Click here for file

Additional file 3 **Transcription regulator genes in cancer and normal network**. It shows the transcription regulator genes in cancer and normal network. Click here for file

Additional file 4 **functional annotations and their p-values of dependent genes affected by transcription regulatory genes in cancer network**. We filtered the functional annotations results at least 3 members in each functional category and P-value < 0.05 with Bonferroni correction and FDR<0.25 using DAVID online toolkit. Click here for file

Additional file 5 **the enrichment canonical pathways and their p-values in cancer network**. We filtered the indeed functional enrichment canonical pathways of the overlap of the gene set in our networks with at least 3 members in each functional category and P value < 0.05 using GSEA online toolkit. Click here for file

Additional file 6 **The genes are belong to the enrichment canonical pathways in cancer network**. The genes are belong to the functional enrichment pathways in cancer network. Click here for file

Additional file 7 **the p-value of pair of genes involved in the figure **12 and 13. It shows the p-value of pair of genes involved in the figure 12 and 13. The column "Co-expressed genes: denotes dependent genes (Dgs) of transcription regulator genes (Tgs). The column "TF" means transcription regulator genes (Tgs). The column "d-separated genes" denotes the minimum d-separated genes between Co-expressed genes and TF. The column "P-value" means the statistical p-value calculated by conditional independency testing in cancer and normal network. Click here for file

Additional file 8 **the conditional impendence testing results between without/with Bonferroni correction**. It shows the conditional impendence testing results of two genes between without/with Bonferroni correction. The column "Co-expressed genes: denotes dependent genes (Dgs) of transcription regulator genes (Tgs). The column "TF" means transcription regulator genes (Tgs). The column "d-separated genes" denotes the minimum d-separated genes between Co-expressed genes and TF. The column "P-value" means the statistical p-value calculated by conditional independency testing. The column "with Bonferroni correction" and "without Bonferroni correction" show the multiple testing to verify the result dependent on d-separated genes and only with a significant value 0.05 to verify the results. Click here for file

Additional file 9 **Feedback loop network motifs in cancer and normal network**. It shows the genes and their relations involved in the feedback loop network motifs in cancer and normal network. Tg denotes transcription regulatory gene and the sign (+, -) mean the activation or inhibition of pair of genes. Click here for file

Additional file 10 **Transcription regulator genes in DOR network motifs in cancer and normal networks**. It shows only the transcription regulator genes involved in the dense overlapping regulons network motifs in cancer and normal network. Click here for file

Additional file 11 **Transcription regulator genes in FFL network motifs in cancer and normal networks**. It shows only the transcription regulator genes in the feed forward loop network motifs in cancer and normal network. Tg denotes the first transcription regulatory gene and co-Tg means the second transcription regulatory gene which affects the dependent genes with the first Tg. Click here for file

Additional file 12 **Evaluated result of Transcription regulator genes with respect to prostate cancer**. There are three labels which we used to evaluate each transcription regulator gene (Tg) implicated in cancer network: Match, Possible, and Not-related. "Match" means if Tg is published in the literature and reported an important role to affect the cancer. "Possible" means one of "Other genes possibly implicated in cancer" listed in Atlas of Genetics and Cytogenetics in Oncology and Haematology. If no information about the relationship of Tg and a cancer is labeled as "Not-related". Click here for file

Additional file 13 **Verifications based on literature reports of each Transcription regulator genes in cancer network**. It shows the Verifications based on literature reports of each transcription regulator genes in cancer network and label the strength of those genes belong to the prostate cancer. Click here for file
