# Supplementary material for: Identifying significant genetic regulatory networks in the prostate cancer from microarray data based on transcription factor analysis and conditional independency
Source: BMC Med Genomics. 2009 Dec 21;2:70. doi: 10.1186/1755-8794-2-70 (PMC2805685; doi:10.1186/1755-8794-2-70)
Supplement: Additional file 1 — KNN imputed ratio. Table shows the maximal values can be imputed by KNN algorithm and the number of values is exactly imputed in each microarry data. The imputed ratio is the proportion of the real imputed genes to the maximal genes. The less the imputed ratio is, the more imputed data similar to real experiment data. The result shows that the imputed ratios of the microarray data are all less than 50%, it seems to be reasonable to assume the imputed dataset is good enough to analyze the gene regulatory network. [file 1755-8794-2-70-S1.PDF]

	Cancer	Normal
Maximum imputed values in the microarray data	155820	92192
Real imputed values in the microarray data	53636	31086
Imputed ratio	34%	34%
