# Supplementary material for: Identifying significant genetic regulatory networks in the prostate cancer from microarray data based on transcription factor analysis and conditional independency
Source: BMC Med Genomics. 2009 Dec 21;2:70. doi: 10.1186/1755-8794-2-70 (PMC2805685; doi:10.1186/1755-8794-2-70)
Supplement: Additional file 2 — All pair shortest path in cancer and normal network. We use all pairs shortest path Dijkstra's algorithm to detect the length of any one of gene link to other genes in cancer and normal network. [file 1755-8794-2-70-S2.PDF]

Category	Cancer network						Normal network					
The length of shortest path	1	2	3	4	5		1	2	3	4	5	6
probability	0.10%	34.6%	31.4%	33.8%	0.07%		0.11%	16.6%	12.8%	68.7%	1.36%	0.45%
