# Supplementary material for: Identifying significant genetic regulatory networks in the prostate cancer from microarray data based on transcription factor analysis and conditional independency
Source: BMC Med Genomics. 2009 Dec 21;2:70. doi: 10.1186/1755-8794-2-70 (PMC2805685; doi:10.1186/1755-8794-2-70)
Supplement: Additional file 3 — Transcription regulator genes in cancer and normal network. It shows the transcription regulator genes in cancer and normal network. [file 1755-8794-2-70-S3.PDF]

Transcription regulator genes in cancer network			Transcription regulator genes in normal network		
SREBF1	EP300	HSF2	POU2F1	DDIT3	NFYB
NFKB1	RUNX1	STAT1	TBP	CUTL1	RUNX1
STAT3	STAT6	TBP	XBP1	SRF	SP1
CUTL1	PBX1	ATF2	NR3C1	REL	NR2F1
POU2F1	YY1	E2F3	STAT1	MEF2A	RELA
RELA	SP1	REL	E2F4	ATF2	MYC
GATA3	MYC	PRL	HSF2	ARNT	MAX
TCF4	ARNT	E2F5	YY1	EGR2	
JUN	MAX	NR2F2			
MEF2A	DDIT3	NR2F1			
NFYB	EGR2	EGR1			
NR3C1	SRF	XBP1			
