# Supplementary material for: Identifying significant genetic regulatory networks in the prostate cancer from microarray data based on transcription factor analysis and conditional independency
Source: BMC Med Genomics. 2009 Dec 21;2:70. doi: 10.1186/1755-8794-2-70 (PMC2805685; doi:10.1186/1755-8794-2-70)
Supplement: Additional file 4 — functional annotations and their p-values of dependent genes affected by transcription regulatory genes in cancer network. We filtered the functional annotations results at least 3 members in each functional category and P-value < 0.05 with Bonferroni correction and FDR<0.25 using DAVID online toolkit. [file 1755-8794-2-70-S4.PDF]

Term	*PValue*
GO:0044238~primary metabolic process	*2.28E-20*
GO:0008152~metabolic process	*3.29E-18*
GO:0044237~cellular metabolic process	*3.78E-18*
GO:0043283~biopolymer metabolic process	*1.05E-13*
GO:0043170~macromolecule metabolic process	*4.02E-12*
GO:0065007~biological regulation	*6.05E-12*
GO:0050789~regulation of biological process	*1.39E-11*
GO:0050794~regulation of cellular process	*1.86E-11*
GO:0048522~positive regulation of cellular process	*1.16E-10*
GO:0048518~positive regulation of biological process	*4.30E-09*
GO:0043412~biopolymer modification	*5.03E-09*
GO:0006139~nucleobase, nucleoside, nucleotide and nucleic acid metabolic process	*9.60E-09*
GO:0006464~protein modification process	*1.31E-08*
GO:0009987~cellular process	*2.09E-08*
GO:0043687~post-translational protein modification	*2.96E-08*
GO:0016071~mRNA metabolic process	*7.06E-08*
GO:0006396~RNA processing	*8.23E-08*
GO:0010467~gene expression	*9.99E-08*
GO:0044267~cellular protein metabolic process	*1.98E-07*
GO:0044260~cellular macromolecule metabolic process	*2.43E-07*
GO:0016070~RNA metabolic process	*3.54E-07*
GO:0019538~protein metabolic process	*5.58E-07*
GO:0006397~mRNA processing	*6.02E-07*
GO:0008380~RNA splicing	*6.05E-07*
GO:0007242~intracellular signaling cascade	*1.29E-06*
GO:0045935~positive regulation of nucleobase, nucleoside, nucleotide and nucleic acid metabolic process	*1.41E-06*
GO:0045941~positive regulation of transcription	*2.17E-06*
GO:0048523~negative regulation of cellular process	*2.79E-06*
GO:0043123~positive regulation of I-kappaB kinase/NF-kappaB cascade	*3.06E-06*
GO:0009893~positive regulation of metabolic process	*3.10E-06*
GO:0033036~macromolecule localization	*3.81E-06*
GO:0043122~regulation of I-kappaB kinase/NF-kappaB cascade	*4.88E-06*
GO:0015031~protein transport	*5.82E-06*
GO:0031325~positive regulation of cellular metabolic process	*6.12E-06*
GO:0048519~negative regulation of biological process	*6.28E-06*

GO:0045184~establishment of protein localization	*1.69E-05*
GO:0016568~chromatin modification	*2.07E-05*
GO:0044265~cellular macromolecule catabolic process	*2.62E-05*
GO:0008219~cell death	*2.89E-05*
GO:0016265~death	*2.89E-05*
GO:0008104~protein localization	*3.60E-05*
GO:0016192~vesicle-mediated transport	*4.39E-05*
GO:0044248~cellular catabolic process	*4.82E-05*
GO:0012501~programmed cell death	*6.06E-05*
GO:0007249~I-kappaB kinase/NF-kappaB cascade	*6.63E-05*
GO:0000375~RNA splicing, via transesterification reactions	*8.87E-05*
GO:0000377~RNA splicing, via transesterification reactions with bulged adenosine as nucleophile	*8.87E-05*
GO:0000398~nuclear mRNA splicing, via spliceosome	*8.87E-05*
GO:0006915~apoptosis	*9.66E-05*
