# Supplementary material for: Identifying significant genetic regulatory networks in the prostate cancer from microarray data based on transcription factor analysis and conditional independency
Source: BMC Med Genomics. 2009 Dec 21;2:70. doi: 10.1186/1755-8794-2-70 (PMC2805685; doi:10.1186/1755-8794-2-70)
Supplement: Additional file 5 — the enrichment canonical pathways and their p-values in cancer network. We filtered the indeed functional enrichment canonical pathways of the overlap of the gene set in our networks with at least 3 members in each functional category and P value < 0.05 using GSEA online toolkit. [file 1755-8794-2-70-S5.PDF]

Term name	*p value*
HSA04080_NEUROACTIVE_LIGAND_RECEPTOR_INTERACTION	*1.93E-39*
GPCRDB_CLASS_A_RHODOPSIN_LIKE	*1.98E-35*
HSA04060_CYTOKINE_CYTOKINE_RECEPTOR_INTERACTION	*1.33E-31*
HSA04810_REGULATION_OF_ACTIN_CYTOSKELETON	*4.99E-25*
HSA01430_CELL_COMMUNICATION	*1.10E-24*
HSA04010_MAPK_SIGNALING_PATHWAY	*9.13E-21*
HSA04510_FOCAL_ADHESION	*4.96E-19*
HSA04514_CELL_ADHESION_MOLECULES	*9.00E-18*
CALCIUM_REGULATION_IN_CARDIAC_CELLS	*2.69E-16*
HSA01030_GLYCAN_STRUCTURES_BIOSYNTHESIS_1	*4.47E-16*
HSA04650_NATURAL_KILLER_CELL_MEDIATED_CYTOTOXICITY	*9.97E-16*
HSA04020_CALCIUM_SIGNALING_PATHWAY	*1.06E-15*
HSA04630_JAK_STAT_SIGNALING_PATHWAY	*2.87E-15*
HSA04360_AXON_GUIDANCE	*5.88E-15*
INTEGRIN_MEDIATED_CELL_ADHESION_KEGG	*1.70E-14*
PEPTIDE_GPCRS	*3.61E-14*
HSA04670_LEUKOCYTE_TRANSENDOTHELIAL_MIGRATION	*5.20E-14*
HSA04530_TIGHT_JUNCTION	*6.84E-13*
HSA04640_HEMATOPOIETIC_CELL_LINEAGE	*7.62E-13*
SMOOTH_MUSCLE_CONTRACTION	*8.42E-13*
HSA04512_ECM_RECEPTOR_INTERACTION	*1.22E-12*
RIBOSOMAL_PROTEINS	*6.69E-12*
HSA04310_WNT_SIGNALING_PATHWAY	*1.81E-11*
HSA04660_T_CELL_RECEPTOR_SIGNALING_PATHWAY	*2.46E-11*
GPCRDB_OTHER	*3.46E-11*
HSA04110_CELL_CYCLE	*3.84E-11*
CALCINEURIN_NF_AT_SIGNALING	*1.82E-10*
HSA04742_TASTE_TRANSDUCTION	*1.87E-10*
HSA00500_STARCH_AND_SUCROSE_METABOLISM	*2.20E-10*
HSA00230_PURINE_METABOLISM	*2.73E-10*
HSA04612_ANTIGEN_PROCESSING_AND_PRESENTATION	*3.42E-10*
HSA03010_RIBOSOME	*4.16E-10*
BREAST_CANCER_ESTROGEN_SIGNALING	*5.58E-10*
G_PROTEIN_SIGNALING	*6.27E-10*
HSA04115_P53_SIGNALING_PATHWAY	*1.12E-09*
CELL_CYCLE_KEGG	*2.39E-09*

HSA04350_TGF_BETA_SIGNALING_PATHWAY	*2.39E-09*
ST_INTEGRIN_SIGNALING_PATHWAY	*2.85E-09*
HSA04910_INSULIN_SIGNALING_PATHWAY	*2.88E-09*
HSA00150_ANDROGEN_AND_ESTROGEN_METABOLISM	*1.33E-08*
HSA00980_METABOLISM_OF_XENOBIOTICS_BY_CYTOCHROME_P450	*1.73E-08*
HSA03320_PPAR_SIGNALING_PATHWAY	*1.73E-08*
HSA04620_TOLL_LIKE_RECEPTOR_SIGNALING_PATHWAY	*2.42E-08*
HSA04540_GAP_JUNCTION	*2.95E-08*
HSA04730_LONG_TERM_DEPRESSION	*3.47E-08*
HSA05220_CHRONIC_MYELOID_LEUKEMIA	*3.47E-08*
G1_TO_S_CELL_CYCLE_REACTOME	*8.76E-08*
HSA00190_OXIDATIVE_PHOSPHORYLATION	*9.36E-08*
ST_T_CELL_SIGNAL_TRANSDUCTION	*1.15E-07*
HSA04012_ERBB_SIGNALING_PATHWAY	*1.34E-07*
