# Supplementary material for: Identifying significant genetic regulatory networks in the prostate cancer from microarray data based on transcription factor analysis and conditional independency
Source: BMC Med Genomics. 2009 Dec 21;2:70. doi: 10.1186/1755-8794-2-70 (PMC2805685; doi:10.1186/1755-8794-2-70)
Supplement: Additional file 6 — The genes are belong to the enrichment canonical pathways in cancer network. The genes are belong to the functional enrichment pathways in cancer network. [file 1755-8794-2-70-S6.PDF]

Pathway	Genes
HSA04514_CELL_ADHESION_MOLECULES	CD40, F11R, JAM3, ESAM, HLA-DMB, HLA-DPA1, HLA-DPB1, HLA-DQB2, PVRL3, CNTNAP1, GLG1, NRXN2
SMOOTH_MUSCLE_CONTRACTION	EDG2, PRKACB, NFKB1, ATF2, CAMK2D, CALM1, ADCY7, ITPR1, GNAQ, PRKCH, SFN, PRKAR1A, PRKD1, GNG2, RGS1, RGS2, SP1, PDE4B, CRH, ACTA2, ETS2, CNN1, GABPA
HSA04110_CELL_CYCLE	TGFB1, SFN, EP300, CREBBP, SMAD2, CDKN1B, CDK2, E2F3, ORC1L, ORC4L, CDC25A, PRKDC, BUB1B, CDC14B, YWHAZ, YWHAG
HSA00150_ANDROGEN_AND_ESTROGEN_METABOLISM	PRMT3, PRMT5, HSD17B8, STS
HSA04620_TOLL_LIKE_RECEPTOR_SIGNALING_PATHWAY	TNF, CD40, IL12A, PIK3CD, AKT3, MAPK9, MAPK8, MAP2K3, MAPK14, MAPK13, NFKB1, RIPK1, TRAF3, MYD88, IRAK1, IKBKE, TICAM2
HSA03320_PPAR_SIGNALING_PATHWAY	SORBS1, PPARA, LPL, ACOX1, CYP27A1, SCP2, DBI, SLC27A6
HSA04012_ERBB_SIGNALING_PATHWAY	EGFR, RAF1, KRAS, PIK3CD, AKT3, MAPK9, MAPK8, CAMK2D, ERBB3, NCK2, NCK1, CDKN1B, EIF4EBP1, HBEGF
HSA04115_P53_SIGNALING_PATHWAY	SFN, BID, CDK2, GTSE1, RCHY1, SESN1
HSA04310_WNT_SIGNALING_PATHWAY	MAPK9, MAPK8, PRKACB, PPP3CB, PPP3CA, CHP,

	CAMK2D, PLCB4, EP300, CREBBP, SMAD2, CTBP1, FZD1, FZD3, FZD7, BTRC, TCF7L2, FZD4, CSNK1A1, PSEN1, LRP5, LRP6, MMP7, SFRP2, TBL1X
