# Supplementary material for: Identifying significant genetic regulatory networks in the prostate cancer from microarray data based on transcription factor analysis and conditional independency
Source: BMC Med Genomics. 2009 Dec 21;2:70. doi: 10.1186/1755-8794-2-70 (PMC2805685; doi:10.1186/1755-8794-2-70)
Supplement: Additional file 7 — the p-value of pair of genes involved in the figure 12 and 13. It shows the p-value of pair of genes involved in the figure 12 and 13. The column "Co-expressed genes: denotes dependent genes (Dgs) of transcription regulator genes (Tgs). The column "TF" means transcription regulator genes (Tgs). The column "d-separated genes" denotes the minimum d-separated genes between Co-expressed genes and TF. The column "P-value" means the statistical p-value calculated by conditional independency testing in cancer and normal network. [file 1755-8794-2-70-S7.PDF]

TF	*Co-expressed genes*	*P-value (cancer)*	*P-value (normal)*	*TF*	*Co-expressed genes*	*P-value (cancer)*	*P-value (normal)*
RUNX1	*OSBPL6*	*0.009629686*	*0.029407425*	*CUTL1*	*RRBP1*	*0.043003622*	*0.04254763*
RUNX1	*LETMD1*	*0.008547106*	*0.011108984*	*CUTL1*	*PEX10*	*0.00679679*	*0.027149398*
RUNX1	*ERCC2*	*0.0011102234*	*0.034235645*	*CUTL1*	*IMP3*	*0.010450561*	*0.04254763*
RELA	*LPPR2*	*4.5447328E-4*	*0.032572273*	*CUTL1*	*GSS*	*0.007401*	*0.04254763*
POU2F1	*IQCE*	*0.0058710147*	*0.024269056*	*CUTL1*	*BCAT2*	*1.01E-05*	*0.027149398*
POU2F1	*DIO2*	*0.013497373*	*0.04597115*	*CUTL1*	*SCAMP2*	*0.032925967*	*0.04254763*
NR3C1	*CCNB3*	*7.935596E-4*	*0.03330131*	*ATF2*	*SRP54*	*1.7838758E-4*	*0.042158585*
NFYB	*UBP1*	*8.842647E-5*	*0.034235645*	*ATF2*	*SCAMP2*	*0.0039149094*	*0.024754744*
MYC	*CDKN1C*	*1.4000891E-4*	*0.014514497*	*ATF2*	*SAA2*	*0.036163047*	*0.037441906*
MAX	*WASF2*	*0.0017279614*	*0.024358286*	*ATF2*	*MOCS2*	*1.4079492E-4*	*0.024754744*
MAX	*MAP1A*	*0.0047236546*	*0.029633071*	*ATF2*	*METTL4*	*0.0074364496*	*0.028129498*
HSF2	*TMEM111*	*0.044496004*	*0.016043136*	*ATF2*	*LRRC40*	*0.0050933417*	*0.042158585*
HSF2	*PPP6C*	*0.009629684*	*0.016043136*	*ATF2*	*ITGAV*	*2.1689934E-4*	*0.042158585*
HSF2	*MINA*	*0.025646118*	*0.038709*	*ATF2*	*ADSS*	*0.0033400601*	*0.024754744*
HSF2	*LRRC56*	*0.00419005*	*0.016043136*	*YY1*	*ZNF161*	*0.04080116*	*0.027149398*
HSF2	*GNPAT*	*0.005439883*	*0.006876428*	*STAT1*	*PPP1R9A*	*0.003285322*	*0.037425086*
HSF2	*DLG4*	*0.004241443*	*0.01278562*	*STAT1*	*MAN2A1*	*0.010591449*	*0.049536288*
CUTL1	*USF2*	*0.007400838*	*0.04254763*	*SP1*	*RCP9*	*0.02644963745*	*0.026796577*

TF	*Co-expressed genes*	*d-separated genes*	*P-value*
MYC	*CDKN1C*	*SP1*	*0.04*
MAX	*WASF2*	*ATF2*	*0.035*
ATF2	*SCAMP2*	*SP1*	*0.00288*
MAX	*MAPIA*	*ATF2*	*0.358.*
CUTL1	*USF2*	*NFKB1*	*0.035*
SP1	*RCP9*	*ATF2*	*0.039*

TF	*Co-expressed genes*	*P-value (cancer)*	*P-value (normal)*	*TF*	*Co-expressed genes*	*P-value (cancer)*	*P-value (normal)*
RUNX1	*THBS2*	*0.004105856*	*0.037425086*	*POU2F1*	*FCGR3A*	*0.001333*	*0.02539607*
RUNX1	*SLIT2*	*0.0052668615*	*0.007161761*	*MAX*	*ZNF673*	*8.17E-06*	*4.05E-04*
RUNX1	*RDH10*	*5.163008E-6*	*0.010763738*	*MAX*	*ANKZF1*	*0.005743*	*0.012005*
RUNX1	*PTGER4*	*0.03749339*	*0.007170431*	*HSF2*	*ZNF566*	*0.005777*	*0.046002876*
RELA	*SHB*	*0.0012596726*	*0.030953176*	*HSF2*	*SULT1E1*	*0.031510357*	*0.002429*
RELA	*ORF1-FL49*	*0.015224895*	*0.04061879*	*HSF2*	*PSRC2*	*2.16E-04*	*0.03948673*
RELA	*EHD4*	*1.5573391E-4*	*8.013012E-4*	*HSF2*	*CPXM2*	*0.015615404*	*0.016043*
RELA	*DCTN1*	*0.0013332678*	*0.016399333*	*HSF2*	*C1ORF71*	*7.94E-04*	*0.03948673*
POU2F1	*GRLF1*	*9.7548956E-4*	*0.032202344*	*EGR2*	*RXRA*	*0.038187733*	*0.002096*
CUTL1	*RAN*	*0.0389409*	*0.00201517*	*YY1*	*CCNDBP1*	*0.021759933*	*0.04254763*
CUTL1	*PIK3R3*	*0.03575455*	*0.0062399227*	*YY1*	*ARHGEF6*	*0.017831*	*0.02347*
CUTL1	*PERP*	*0.0013208861*	*0.023313634*	*TBP*	*VPS4A*	*0.02329899*	*0.046382897*
CUTL1	*CEPT1*	*0.008115815*	*0.005760788*	*TBP*	*UBE2B*	*4.51E-05*	*0.011937*
CUTL1	*CCDC6*	*0.00501*	*0.01076*	*TBP*	*PGPEP1*	*0.023973*	*0.02331*

		22477	*3738*			*072*	*4*
CUTL1	*ATP2B1*	*0.0389409*	*0.02346984*	*TBP*	*OAS2*	*6.82E-04*	*0.009006*
CUTL1	*ACBD3*	*0.026622215*	*0.015942862*	*TBP*	*C14ORF92*	*0.003269*	*0.016128*
YY1	*HDGFRP3*	*0.04846581*	*0.027040651*	*TBP*	*ALCAM*	*0.011275*	*0.016128*
YY1	*DHX29*	*0.007295*	*0.027149398*				

TF	***Co-expressed genes***	***d-separated genes***	***P-value***
HSF2	*PSRC2*	*NFKB1*	*0.01124*
EGR2	*RXRA*	*E2F3*	*0.00868*
TBP	*VPS4A*	*ATF2*	*0.013216*
TBP	*PGPEP1*	*ATF2*	*0.01876*
TBP	*OAS2*	*PBX1*	*0.01808*
TBP	*C14ORF92*	*PBX1*	*0.019169*
TBP	*ALCAM*	*ATF2*	*0.03971*
