# Supplementary material for: Identifying significant genetic regulatory networks in the prostate cancer from microarray data based on transcription factor analysis and conditional independency
Source: BMC Med Genomics. 2009 Dec 21;2:70. doi: 10.1186/1755-8794-2-70 (PMC2805685; doi:10.1186/1755-8794-2-70)
Supplement: Additional file 8 — the conditional impendence testing results between without/with Bonferroni correction. It shows the conditional impendence testing results of two genes between without/with Bonferroni correction. The column "Co-expressed genes: denotes dependent genes (Dgs) of transcription regulator genes (Tgs). The column "TF" means transcription regulator genes (Tgs). The column "d-separated genes" denotes the minimum d-separated genes between Co-expressed genes and TF. The column "P-value" means the statistical p-value calculated by conditional independency testing. The column "with Bonferroni correction" and "without Bonferroni correction" show the multiple testing to verify the result dependent on d-separated genes and only with a significant value 0.05 to verify the results. [file 1755-8794-2-70-S8.PDF]

Co-expressed genes	TF	d-separated genes	p-value	with Bonferroni correction	without Bonferroni correction (<0.05)
CGI-69	NFKB1	ATF2,E2F3	0.034758394	Remove link	Keep link
DCI	NFKB1	E2F3,STAT6	0.037146364	Remove link	Keep link
MADD	NFKB1	STAT3,ATF2	0.048963897	Remove link	Keep link
MYO18A	NFKB1	ATF2,E2F3	0.034028158	Remove link	Keep link
PSRC2	NFKB1	CUTL1,MAX	0.047850086	Remove link	Keep link
C20ORF11	NFKB1	ATF2,POU2F1	0.026820036	Remove link	Keep link
TGFB1I1	NFKB1	SP1,CUTL1	0.031692074	Remove link	Keep link
NPAS3	STAT3	ATF2,STAT6	0.032231668	Remove link	Keep link
P2RY5	STAT3	STAT6,ATF2	0.032585746	Remove link	Keep link
TCEA1	STAT3	ATF2,E2F3	0.040714405	Remove link	Keep link
TIA1	STAT3	ATF2,STAT6	0.032231668	Remove link	Keep link
XRN1	STAT3	SP1,STAT1	0.042630634	Remove link	Keep link
CNTNAP3	STAT3	STAT6,STAT1	0.042272742	Remove link	Keep link
LIX1L	STAT3	STAT6,ATF2	0.042064083	Remove link	Keep link
SLC13A1	STAT3	STAT6,SP1	0.04769278	Remove link	Keep link
CLASP1	STAT3	STAT6,SP1	0.047444366	Remove link	Keep link
DKFZP564O0823	STAT3	STAT6,SP1	0.025855848	Remove link	Keep link
NCF1	STAT3	SP1,STAT6	0.037384342	Remove link	Keep link
PDLIM5	STAT3	STAT6,SP1	0.026498566	Remove link	Keep link
PIP5K1B	STAT3	STAT6,SP1	0.033233934	Remove link	Keep link
PTN	STAT3	STAT6,SP1	0.025855848	Remove link	Keep link
SLC15A2	STAT3	STAT6,SP1	0.025855848	Remove link	Keep link
ATAD1	STAT6	ATF2,PBX1	0.036172756	Remove link	Keep link
TGM2	SP1	ATF2,PBX1	0.028081318	Remove link	Keep link
ARL1	SP1	ATF2,PBX1	0.025361594	Remove link	Keep link
ARL8A	SP1	ATF2,E2F5	0.047045113	Remove link	Keep link
ASAH1	SP1	ATF2,PBX1	0.030284059	Remove link	Keep link
C10ORF6	SP1	ATF2,POU2F1	0.040892673	Remove link	Keep link
C21ORF25	SP1	ATF2,PBX1	0.03509841	Remove link	Keep link
C2ORF30	SP1	ATF2,PBX1	0.037629293	Remove link	Keep link
F11R	SP1	ATF2,PBX1	0.035960312	Remove link	Keep link
FAM49A	SP1	ATF2,PBX1	0.048485786	Remove link	Keep link
GPX1	SP1	ATF2,E2F5	0.043087997	Remove link	Keep link
L3MBTL3	SP1	ATF2,PBX1	0.030040905	Remove link	Keep link
MCCC1	SP1	ATF2,PBX1	0.035924652	Remove link	Keep link
MTUS1	SP1	ATF2,PBX1	0.034441156	Remove link	Keep link

PKM2	SP1	ATF2,PBX1	0.042909864	Remove link	Keep link
RXRA	SP1	ATF2,EGR2	0.029142136	Remove link	Keep link
TMEM50B	SP1	ATF2,PBX1	0.035177457	Remove link	Keep link
TWSG1	SP1	ATF2,PBX1	0.034520398	Remove link	Keep link
VPS39	SP1	ATF2,E2F3	0.028747065	Remove link	Keep link
BIN1	SP1	ATF2,E2F3	0.027942407	Remove link	Keep link
MGC5139	SP1	ATF2,POU2F1	0.035481135	Remove link	Keep link
C20ORF11	SP1	ATF2,POU2F1	0.03530549	Remove link	Keep link
C22ORF16	SP1	E2F3,ATF2	0.043394039	Remove link	Keep link
KLHL12	SP1	ATF2,PBX1	0.034584465	Remove link	Keep link
MRPS26	SP1	ATF2,E2F3	0.047871043	Remove link	Keep link
RASSF1	SP1	ATF2,POU2F1	0.044154094	Remove link	Keep link
SSBP3	SP1	ATF2,POU2F1	0.026802463	Remove link	Keep link
THADA	SP1	ATF2,TCF4	0.039663078	Remove link	Keep link
FLJ37453	SP1	ATF2,POU2F1	0.047088157	Remove link	Keep link
GBP2	SP1	ATF2,PBX1	0.038152849	Remove link	Keep link
TBC1D20	SP1	ATF2,PBX1	0.034584465	Remove link	Keep link
MTMR6	SP1	ATF2,POU2F1	0.034580645	Remove link	Keep link
TRPM4	SP1	ATF2,MYC	0.049702934	Remove link	Keep link
VPS4A	SP1	ATF2,PBX1	0.046107476	Remove link	Keep link
FLJ11171	SP1	ATF2,PBX1	0.040427977	Remove link	Keep link
PPM1B	SP1	ATF2,MYC	0.026980104	Remove link	Keep link
IQGAP2	SP1	ATF2,PBX1	0.03509841	Remove link	Keep link
LY6G5B	SP1	ATF2,PBX1	0.039602658	Remove link	Keep link
KIF22	SP1	ATF2,POU2F1	0.037442441	Remove link	Keep link
NUMB	SP1	ATF2,POU2F1	0.034998297	Remove link	Keep link
ZNF259	SP1	ATF2,E2F3	0.039328621	Remove link	Keep link
TXNDC4	XBP1	MAX,PBX1	0.027264376	Remove link	Keep link
KCNS3	HSF2	REL,MEF2A	0.049827627	Remove link	Keep link
ZNF740	MAX	ATF2,REL	0.041172641	Remove link	Keep link
FBXO8	STAT1	ATF2,SP1	0.049125323	Remove link	Keep link
FUT1	ARNT	E2F3,ATF2	0.026975824	Remove link	Keep link
PPM1F	ARNT	SP1,PBX1	0.027229321	Remove link	Keep link
VPS37A	ARNT	PBX1,SP1	0.039798603	Remove link	Keep link
C2ORF30	ATF2	SP1,PBX1	0.02653904	Remove link	Keep link
DEDD2	ATF2	SP1,TBP	0.045183251	Remove link	Keep link
FAM49A	ATF2	SP1,PBX1	0.030891136	Remove link	Keep link

KLF3	ATF2	SP1,E2F3	0.038884728	Remove link	Keep link
LCOR	ATF2	TBP,PBX1	0.044878525	Remove link	Keep link
MRPL53	ATF2	E2F3,TBP	0.041917041	Remove link	Keep link
WDR73	ATF2	TBP,EP300	0.046299045	Remove link	Keep link
NPL	ATF2	STAT1,SP1	0.027601709	Remove link	Keep link
ARFGEF1	ATF2	SP1,STAT1	0.049062976	Remove link	Keep link
DYNC1I2	ATF2	NFYB,PBX1	0.032546804	Remove link	Keep link
KIAA1219	ATF2	SP1,GATA3	0.048831537	Remove link	Keep link
SLC15A2	ATF2	PBX1,SP1	0.048907629	Remove link	Keep link
C9ORF97	ATF2	E2F3,TBP	0.041917041	Remove link	Keep link
SDF2	ATF2	PBX1,YY1	0.043663184	Remove link	Keep link
SLC30A6	ATF2	E2F3,PBX1	0.02662567	Remove link	Keep link
AAAS	ATF2	YY1,SP1	0.03050642	Remove link	Keep link
HNMT	MYC	POU2F1,SP1	0.046628035	Remove link	Keep link
FAM49A	PBX1	ATF2,SP1	0.02922921	Remove link	Keep link
FNTA	PBX1	ATF2,SP1	0.027396865	Remove link	Keep link
HOOK3	PBX1	ATF2,SP1	0.031699895	Remove link	Keep link
MGC14376	STAT3	STAT6,STAT1,ATF2	0.022790765	Remove link	Keep link
CALD1	STAT3	STAT6,SP1,ATF2	0.048810133	Remove link	Keep link
CDS1	SP1	ATF2,E2F5,PBX1	0.034273254	Remove link	Keep link
CSK	ATF2	STAT1,TBP,PBX1	0.035092739	Remove link	Keep link
DAZAP2	ATF2	E2F3,PBX1,ARNT	0.049783554	Remove link	Keep link
