# Supplementary material for: Identifying significant genetic regulatory networks in the prostate cancer from microarray data based on transcription factor analysis and conditional independency
Source: BMC Med Genomics. 2009 Dec 21;2:70. doi: 10.1186/1755-8794-2-70 (PMC2805685; doi:10.1186/1755-8794-2-70)
Supplement: Additional file 9 — Feedback loop network motifs in cancer and normal network. It shows the genes and their relations involved in the feedback loop network motifs in cancer and normal network. Tg denotes transcription regulatory gene and the sign (+, -) mean the activation or inhibition of pair of genes. [file 1755-8794-2-70-S9.PDF]

Feedback loop network motifs in cancer network							Feedback loop network motifs in normal network					
Tg		Tg		Tg			Tg		Tg		Tg	
NFKB1	+	STAT6	+	STAT3	+		POU2F1	+	TBP	+	RUNX1	+
NFKB1	+	STAT6	+	ATF2	+		TBP	+	RUNX1	+	MAX	+
NFKB1	+	EGR1	-	E2F3	-		TBP	+	RUNX1	+	YY1	+
STAT1	+	STAT3	+	ATF2	+		RUNX1	+	TBP	+	POU2F1	+
STAT1	+	ATF2	+	STAT3	+		MAX	+	RUNX1	+	TBP	+
STAT3	+	NFKB1	+	STAT6	+		MAX	+	RUNX1	+	YY1	+
STAT3	+	STAT1	+	ATF2	+		YY1	+	RUNX1	+	TBP	+
STAT3	+	ATF2	+	STAT1	+		YY1	+	RUNX1	+	MAX	+
STAT3	+	ATF2	-	E2F3	-							
STAT3	+	ATF2	+	SP1	+							
STAT3	+	EGR1	-	E2F3	-							
STAT6	+	STAT3	+	NFKB1	+							
STAT6	+	ATF2	+	NFKB1	+							
STAT6	+	ATF2	+	MAX	-							
ATF2	+	NFKB1	+	STAT6	+							
ATF2	+	STAT1	+	STAT3	+							
ATF2	+	STAT3	+	STAT1	+							
ATF2	-	E2F3	-	STAT3	+							
ATF2	+	SP1	+	STAT3	+							
ATF2	+	MAX	-	STAT6	+							
E2F3	-	NFKB1	+	EGR1	-							
E2F3	-	STAT3	+	ATF2	-							
E2F3	-	STAT3	+	EGR1	-							
E2F3	-	NR2F2	+	EGR1	-							
SP1	+	STAT3	+	ATF2	+							
MAX	-	STAT6	+	ATF2	+							
NR2F2	+	EGR1	-	E2F3	-							
EGR1	-	E2F3	-	NFKB1	+							
EGR1	-	E2F3	-	STAT3	+							
EGR1	-	E2F3	-	NR2F2	+							
SREBF1	+	STAT6	+	NFKB1	+							
EP300	+	NFKB1	+	STAT6	+							
EP300	+	NFKB1	-	CUTL1	-							
EP300	+	STAT3	+	STAT1	+							
EP300	+	STAT3	+	ATF2	+							

EP300	+	STAT3	-	E2F3	-							
EP300	+	STAT6	+	SREBF1	+							
EP300	+	STAT6	+	NFKB1	+							
EP300	+	SP1	+	ATF2	+							
EP300	+	SP1	+	E2F5	+							
EP300	+	SP1	+	EGR2	+							
NFKB1	+	STAT6	+	SREBF1	+							
NFKB1	+	EGR1	-	E2F3	-							
STAT1	+	NFKB1	+	STAT6	+							
STAT1	+	NFKB1	-	CUTL1	-							
STAT1	+	STAT3	+	ATF2	+							
STAT1	+	STAT3	-	E2F3	-							
STAT1	+	ATF2	+	STAT3	+							
STAT1	+	ATF2	+	TBP	+							
STAT1	+	ATF2	+	PBX1	+							
STAT1	+	ATF2	-	YY1	-							
STAT1	+	ATF2	+	SP1	+							
STAT1	+	EGR1	-	E2F3	-							
STAT3	+	NFKB1	+	STAT6	+							
STAT3	+	NFKB1	-	CUTL1	-							
STAT3	+	STAT1	+	ATF2	+							
STAT3	+	ATF2	+	STAT1	+							
STAT3	+	ATF2	+	TBP	+							
STAT3	+	ATF2	+	PBX1	+							
STAT3	+	ATF2	-	YY1	-							
STAT3	+	ATF2	+	SP1	+							
STAT3	-	E2F3	-	EGR1	-							
STAT3	+	EGR1	-	E2F3	-							
STAT6	+	NFKB1	-	CUTL1	-							
STAT6	+	STAT3	+	STAT1	+							
STAT6	+	STAT3	+	ATF2	+							
STAT6	+	STAT3	-	E2F3	-							
STAT6	+	ATF2	+	STAT1	+							
STAT6	+	ATF2	+	STAT3	+							
STAT6	+	ATF2	+	TBP	+							
STAT6	+	ATF2	+	PBX1	+							
STAT6	+	ATF2	-	YY1	-							

STAT6	+	ATF2	+	SP1	+								
TBP	+	ATF2	+	STAT1	+								
TBP	+	ATF2	+	STAT3	+								
TBP	+	ATF2	+	PBX1	+								
TBP	+	ATF2	-	YY1	-								
TBP	+	ATF2	+	SP1	+								
CUTL1	-	NFKB1	+	STAT6	+								
CUTL1	+	MYC	-	POU2F1	-								
PBX1	+	EP300	+	SP1	+								
PBX1	+	ATF2	+	STAT1	+								
PBX1	+	ATF2	+	STAT3	+								
PBX1	+	ATF2	+	TBP	+								
PBX1	+	ATF2	-	YY1	-								
PBX1	+	ATF2	+	SP1	+								
PBX1	+	EGR2	+	SP1	+								
ATF2	+	NFKB1	+	STAT6	+								
ATF2	+	NFKB1	-	CUTL1	-								
ATF2	+	STAT1	+	STAT3	+								
ATF2	+	STAT3	+	STAT1	+								
ATF2	+	STAT3	-	E2F3	-								
ATF2	-	E2F3	-	STAT3	-								
ATF2	-	E2F3	-	EGR1	-								
ATF2	+	SP1	+	EP300	+								
ATF2	+	SP1	+	E2F5	+								
ATF2	+	SP1	+	EGR2	+								
ATF2	+	EGR1	-	E2F3	-								
YY1	-	ATF2	+	STAT1	+								
YY1	-	ATF2	+	STAT3	+								
YY1	-	ATF2	+	TBP	+								
YY1	-	ATF2	+	PBX1	+								
YY1	-	ATF2	+	SP1	+								
E2F3	-	NFKB1	+	STAT6	+								
E2F3	-	NFKB1	-	CUTL1	-								
E2F3	-	STAT3	+	STAT1	+								
E2F3	-	STAT3	+	ATF2	+								
RELA	+	SREBF1	+	STAT6	+								
SP1	+	NFKB1	+	STAT6	+								

SP1	+	NFKB1	-	CUTL1	-							
SP1	+	STAT3	+	STAT1	+							
SP1	+	STAT3	+	ATF2	+							
SP1	+	STAT3	-	E2F3	-							
SP1	+	ATF2	+	STAT1	+							
SP1	+	ATF2	+	STAT3	+							
SP1	+	ATF2	+	TBP	+							
SP1	+	ATF2	+	PBX1	+							
SP1	+	ATF2	-	YY1	-							
GATA3	-	EGR2	+	SP1	+							
E2F5	+	SP1	+	EP300	+							
E2F5	+	SP1	+	ATF2	+							
E2F5	+	SP1	+	EGR2	+							
E2F5	+	MYC	-	POU2F1	-							
MAX	-	STAT6	+	SREBF1	+							
MAX	-	STAT6	+	NFKB1	+							
NR2F2	+	EGR1	-	E2F3	-							
EGR2	+	STAT6	+	SREBF1	+							
EGR2	+	STAT6	+	NFKB1	+							
EGR2	+	SP1	+	EP300	+							
EGR2	+	SP1	+	ATF2	+							
EGR2	+	SP1	+	E2F5	+							
EGR1	-	E2F3	-	STAT3	-							
