# Supplementary material for: Identifying significant genetic regulatory networks in the prostate cancer from microarray data based on transcription factor analysis and conditional independency
Source: BMC Med Genomics. 2009 Dec 21;2:70. doi: 10.1186/1755-8794-2-70 (PMC2805685; doi:10.1186/1755-8794-2-70)
Supplement: Additional file 10 — Transcription regulator genes in DOR network motifs in cancer and normal networks. It shows only the transcription regulator genes involved in the dense overlapping regulons network motifs in cancer and normal network. [file 1755-8794-2-70-S10.PDF]

| Transcription regulatory genes of DOR network motifs in cancer network | | | Transcription regulatory genes of DOR network motifs in normal network | | |
|---|---|---|---|---|---|
| TCF4 | SRF | PRL | XBP1 | STAT1 | SRF |
| NR3C1 | NR2F1 | NFYB | SP1 | NR3C1 | NR2F1 |
| MEF2A | DDIT3 | | NFYB | MYC | MEF2A |
| | | | EGR2 | E2F4 | ATF2 |
| | | | ARNT | | |
