# Supplementary material for: Identifying significant genetic regulatory networks in the prostate cancer from microarray data based on transcription factor analysis and conditional independency
Source: BMC Med Genomics. 2009 Dec 21;2:70. doi: 10.1186/1755-8794-2-70 (PMC2805685; doi:10.1186/1755-8794-2-70)
Supplement: Additional file 11 — Transcription regulator genes in FFL network motifs in cancer and normal networks. It shows only the transcription regulator genes in the feed forward loop network motifs in cancer and normal network. Tg denotes the first transcription regulatory gene and co-Tg means the second transcription regulatory gene which affects the dependent genes with the first Tg. [file 1755-8794-2-70-S11.PDF]

Transcription regulatory genes of FFL network motifs in cancer network		Transcription regulatory genes of FFL network motifs in normal network	
Tg	Co-Tg	Tg	Co-Tg
SREBF1	STAT6	TBP	RUNX1
EP300	NFKB1	TBP	EGR2
EP300	STAT3	RUNX1	MAX
EP300	STAT6	RUNX1	YY1
EP300	SP1	CUTL1	STAT1
EP300	XBP1	CUTL1	RELA
HSF2	MAX	REL	MEF2A
HSF2	NFYB	HSF2	DDIT3
NFKB1	STAT6	HSF2	MEF2A
NFKB1	CUTL1		
NFKB1	EGR1		
RUNX1	NR2F2		
STAT1	NFKB1		
STAT1	STAT3		
STAT1	ATF2		
STAT1	ARNT		
STAT1	EGR1		
STAT1	XBP1		
STAT3	NFKB1		
STAT3	ATF2		
STAT3	E2F3		
STAT3	ARNT		
STAT3	NR2F2		
STAT3	EGR1		
STAT3	XBP1		
STAT6	NFKB1		
STAT6	STAT3		
STAT6	ATF2		
STAT6	NR2F1		
STAT6	XBP1		
TBP	ATF2		
TBP	MEF2A		
CUTL1	NFKB1		
CUTL1	MYC		

PBX1	EP300		
PBX1	ATF2		
PBX1	EGR2		
PBX1	XBP1		
ATF2	NFKB1		
ATF2	PBX1		
ATF2	YY1		
ATF2	E2F3		
ATF2	SP1		
ATF2	TCF4		
ATF2	MAX		
ATF2	NFYB		
ATF2	EGR1		
ATF2	XBP1		
POU2F1	MYC		
POU2F1	NR3C1		
YY1	MAX		
YY1	DDIT3		
E2F3	NFKB1		
E2F3	STAT3		
E2F3	ARNT		
E2F3	NR2F2		
E2F3	NR2F1		
E2F3	EGR1		
E2F3	XBP1		
RELA	SREBF1		
SP1	EP300		
SP1	NFKB1		
SP1	STAT3		
SP1	ATF2		
SP1	ARNT		
SP1	E2F5		
SP1	NR2F2		
SP1	EGR2		
REL	JUN		
REL	MAX		
REL	SRF		

GATA3	EGR2		
MYC	POU2F1		
ARNT	TCF4		
ARNT	DDIT3		
E2F5	SP1		
E2F5	MYC		
JUN	REL		
JUN	SRF		
MAX	STAT6		
MAX	NFYB		
MAX	SRF		
MAX	XBP1		
EGR2	STAT6		
EGR2	STAT6		
EGR2	SP1		
EGR1	E2F3		
