# Supplementary material for: Identifying significant genetic regulatory networks in the prostate cancer from microarray data based on transcription factor analysis and conditional independency
Source: BMC Med Genomics. 2009 Dec 21;2:70. doi: 10.1186/1755-8794-2-70 (PMC2805685; doi:10.1186/1755-8794-2-70)
Supplement: Additional file 12 — Evaluated result of Transcription regulator genes with respect to prostate cancer. There are three labels which we used to evaluate each transcription regulator gene (Tg) implicated in cancer network: Match, Possible, and Not-related. "Match" means if Tg is published in the literature and reported an important role to affect the cancer. "Possible" means one of "Other genes possibly implicated in cancer" listed in Atlas of Genetics and Cytogenetics in Oncology and Haematology. If no information about the relationship of Tg and a cancer is labeled as "Not-related". [file 1755-8794-2-70-S12.PDF]

Transcription regulator gene	Match	Possible	Not-related
SP1	●		
HSF2		●	
MAX		●	
NFYB		●	
RUNX1	●		
STAT1	●		
ATF2		●	
CUTL1		●	
MYC	●		
YY1	●		
POU2F1		●	
NR3C1		●	
RELA		●	
TBP		●	
REL	●		
HSF2		●	
	37.5%	62.5%	0%
