# Supplementary material for: Identifying significant genetic regulatory networks in the prostate cancer from microarray data based on transcription factor analysis and conditional independency
Source: BMC Med Genomics. 2009 Dec 21;2:70. doi: 10.1186/1755-8794-2-70 (PMC2805685; doi:10.1186/1755-8794-2-70)
Supplement: Additional file 13 — Verifications based on literature reports of each Transcription regulator genes in cancer network. It shows the Verifications based on literature reports of each transcription regulator genes in cancer network and label the strength of those genes belong to the prostate cancer. [file 1755-8794-2-70-S13.PDF]

Gene	Literature	Label
SP1	Inhibitory Effects of Quercetin on Proliferation of Prostate Cancer Cells and Activity of Transcription Factor Sp1	Match
HSF2	List in “Other genes possibly implicated in cancer”	Possible
MAX	List in “the annotated genes”	Possible
NFYB	List in “Other genes possibly implicated in cancer”	Possible
RUNX1	Fowler M, Borazanci E, McGhee L, Pylant SW, Willliams BJ, Glass J, Davis JN, and Meyers S. (2006). RUNX1 (AML-1) and RUNX2 (AML-3) cooperate with prostate-derived Ets factor to activate transcription from the PSA upstream regulatory region. J. Cell Biochem. 97:1-17.	Match
STAT1	Novel role of Stat1 in the development of docetaxel resistance in prostate tumor cells. Cancer Chemother Pharmacol. 2006 May 10	Match
ATF2	List in “Other genes possibly implicated in cancer”	Possible
CUTL1	List in “Other genes possibly implicated in cancer”	Possible
MYC	Ellwood-Yen K, Graeber TG, Wongvipat J, et al. Myc-driven murine prostate cancer shares molecular features with human prostate tumors. Cancer Cell 2003;4:223-38.	Match
YY1	YY1: NOVEL PROGNOSTIC FACTOR IN HUMAN PROSTATE CANCER - http://www.research.ucla.edu/tech/ucla04-194.htm	Match
POU2F1	List in “Other genes possibly implicated in cancer”	Possible
NR3C1	List in “Other genes possibly implicated in cancer”	Possible
RELA	List in “the annotated genes”	Possible
TBP	List in “Other genes possibly implicated in cancer”	Possible
REL	Mukhopadhyay NK, Ferdinand AS, et al. Unraveling androgen receptor interactomes by an array-based method: discovery of proto-oncoprotein c-Rel as a negative regulator of androgen receptor.Exp Cell Res; 2006,312(19):3782-95.	Match
HSF2	List in “Other genes possibly implicated in cancer”	Possible
